# Judicial Self‐Governance Index: Towards better understanding of the role of judges in governing the judiciary

**DOI:** 10.1111/rego.12453

**Published:** 2022-01-04

**Authors:** Katarína Šipulová, Samuel Spáč, David Kosař, Tereza Papoušková, Viktor Derka

**Affiliations:** ^1^ Judicial Studies Institute Law Faculty, Masaryk University, Veveří 70, 611 80 Brno Czech Republic; ^2^ Law and Economics Faculty, Universität Bayreuth

**Keywords:** courts, judges, judicial governance, Judicial Self‐Governance, ministerial model

## Abstract

The aim of this article is to introduce a novel view on how to evaluate the share of power held by judges in judicial governance. Its contribution to court administration and the regulation of judges is three‐fold. First, it provides a novel empirically tested conceptualization of judicial governance that includes 60 competences grouped into eight dimensions (ranging from selection and education of judges to case allocation and publication of judicial decisions). Second, it proposes a new Judicial Self‐Governance (JSG) Index that measures how much power domestic judges hold in these eight dimensions of judicial governance. Third, by applying the JSG Index to the longitudinal data for Germany, Italy, Czechia, and Slovakia this article demonstrates that the Judicial Council model is not the only institutional model of judicial governance leading to the empowerment of judges. This means that judges can hold many powers without the existence of judicial councils and even in the Ministry of Justice model.

## 1 Introduction: Reconstructing the understanding of judicial self‐governance and judicial power

Over the past three decades, the growing power of domestic courts has been followed by a rise in Judicial Self‐Governance (“JSG”). The increased influence of courts has prompted the need for a new institutional setup that would ensure a proper balance between judicial independence and judicial accountability (Bunjevac, 2020; Guarnieri, 2004; Ng, 2011; Vapnek, 2013). In attempts to secure this balance, many states across the world have abandoned models dominated by ministries of justice, and instead introduced new forms of judicial governance (Garoupa & Ginsburg, 2009; Hammergren, 2002; Ingram, 2012; Kosař, 2018; Piana, 2010). The regulation of judicial careers, the selection of judges, or court administration has been more and more often vested in the hands of judges. This development was very well demonstrated in postwar Europe, where the majority of countries established judicial councils, either voluntarily (e.g., Italy, France, and the Netherlands) or under the influence of the European Union and the Council of Europe (especially in Central and Eastern Europe [CEE]). To EU candidate countries of CEE judicial councils were not only recommended as the best design, securing principles of judicial independence, separation of powers, and emancipation of judges, but also presented as the “only game in town” (Albrecht & Thomas, 2009; Autheman & Elena, 2004; Piana, 2010). The Council of Europe (CoE) and its interlocutors, such as the Venice Commission, the Consultative Council of European Judges, and the European Network of Councils for the Judiciary, believed that the judicial council model was the cure for all diseases in CEE judiciaries^1^ (Bobek & Kosař, 2014; Parau, 2018). The European Commission wholeheartedly adopted the CoE soft standards during the accession process and imposed it on the EU candidate countries from the CEE in the 2004 and 2007 enlargement waves (Seibert‐Fohr, 2009).

Numerous negative experiences from CEE suggest that these promises have not been fulfilled and judicial councils have failed to keep up with high expectations (Bozhilova, 2007; Kosař, 2018; Parau, 2015; Parau, 2018; Popova, 2010). Not only have they not managed to secure judicial independence (Garoupa & Ginsburg, 2009; Popova, 2020), in some instances, judicial councils have had negative effects (Kosař, 2016; Popova, 2012), isolating the judiciary and helping to foster growing networks of informal practices, such as patronage, nepotism, and corruption (Spáč et al., 2018). Moreover, the recent wave of political backlashes against courts has shown that they are actually very susceptible to political capture (Sadurski, 2019). Despite this development, the CoE and EU bodies, nowadays including the European Court of Human Rights and the Court of Justice of the European Union, still endorse the judicial council model as the superior mode of judicial governance^2^ (Leloup, 2020) and even seem to prevent any undoing or readjustment of such model by developing a “nonregression” principle (Leloup et al., 2020).

Despite the continuing belief of European supranational bodies in judicial councils and despite the considerable resources spent by international organizations in promoting them as the ideal model of governance (Parau, 2015), it seems that judicial councils in fact posit many risks and drawbacks, while their effect remains unclear. First, there is no straightforward relationship between judicial councils and the quality of a judicial system. On the contrary, some countries with a court service (Ireland) or the ministerial model (Germany) have actually scored better in measurements of judicial independence^3^ or efficiency (Czechia and Germany). Some authors even claim that the ministerial model can be more resistant to capture and that it is easier to mobilize civil society against threats from political branches via the Ministry of Justice than against threats from within the judiciary via the judicial council (Kosař, 2016). Second, although judicial councils are seen as the embodiment of judicial empowerment, their composition, powers, and scope of competences differ significantly (Bobek & Kosař, 2014; Castillo‐Ortiz, 2019). In fact, the mere existence of a judicial council does not tell us much about the participation of judges in the regulation and execution of judicial governance. Third, the most recent scholarly works have noticed the increasing importance and agencification of other actors involved in judicial governance, such as chief justices, court presidents, directors of courts, and other regulatory bodies (Kosař & Spáč, 2021; Lurie et al., 2019; Verhoest, 2013).

Reflecting upon these developments, we argue that the differentiation between systems where courts are governed by judicial councils on the one hand and ministries of justice on the other hand, does not sufficiently capture the complexity of judicial governance, nor is it able to reflect the diachronic transformation of the power distribution between politicians, judges, and other actors. We build on the most recent scholarship suggesting that research on JSG needs to look beyond judicial councils and study the role of judges in the governance of courts more generally (Kosař, 2018). As with other fields of regulation (Joradana & Sancho, 2004; Mathieu et al., 2016), we argue that it is important to understand how the judicial governance and power dispersion among multiple actors within is organized. For this reason, we have constructed a novel JSG Index, aimed at capturing this complexity. In doing so, we shift the focus from the grand‐scale model to the question of competence delegation and approach judicial governance as a broad multidimensional concept. This allows us to capture both substantive institutional changes, like the introduction of a new agency (such as the new judicial councils established in the Netherlands and Slovakia), as well as gradual *de jure* changes in the competences of more decentralized actors (such as the agencification of the Director of Courts in Israel; see Lurie et al., 2019).

The purpose of this article is three‐fold. First, we propose a more nuanced understanding of judicial governance and show how it can be measured by constructing the JSG Index. Second, we validate the JSG Index by examining its application in two pairs of countries—Slovakia and Italy, and Czechia and Germany—which allow us to observe the utility of the index with regard to two factors: old versus new members of the EU (within each pair), and a ministerial versus strong judicial council model of judicial governance (across the pairs). In other words, we demonstrate the ability of the JSG Index to capture the *de jure* powers vested in the hands of judges across different models of judicial governance. The first pair, Italy and Slovakia, consists of countries with strong judicial councils with similar sets of competences. The main difference is that Italy is an old EU member state and has an established judicial council, while Slovakia joined the EU only in 2004 and adopted its judicial council only during the EU accession process. The second pair, Germany and Czechia, includes countries that kept the ministerial model of judicial governance. Both Germany, an old EU member state, and Czechia, a new EU member, resisted the establishment of a judicial council, Czechia even in spite of EU pressure during the accession process. Third, explaining the JSG as a multi‐dimensional noncompensatory concept, we discuss how different operationalization strategies affect the results of our JSG Index.

Importantly, the arguments presented in this article are not normative. We refrain from causal inferences and do not offer a definite answer to which model of judicial governance works best. Instead, we argue that the JSG Index ultimately captures the amount of power vested with judges in different institutional setups and permits a careful analysis of the relationship between institutional change and *de jure* empowerment of judges. The results provided by the JSG Index also open an avenue for future research on causality between structural empowerment of judges and outputs of judicial governance: measures of judicial independence and accountability, transparency, and effectiveness of courts, and public trust in the courts. Such results will moreover help to generate a better understanding of institutional reforms of judicial governance and to mitigate political obstacles to achieving a sufficient level of self‐governance in countries where judicial councils, for various reasons, cannot be implemented, for example, due to political deadlock.

The article proceeds as follows. Section 2 discusses the existing scholarship on judicial governance and its measurement. Section 3 presents our conceptualization of JSG. Section 4 discusses the methodological challenges of the JSG Index's construction and explains the choices we made. Then, Section 5 proceeds to test the JSG Index on the longitudinal data for Czechia, Slovakia, Italy, and Germany. Section 6 concludes by setting out the broader repercussions of the JSG Index and possibilities for future research.

## 2 Power to judges? Theories on JSG and its measuring

The judiciary as a third branch of power can wield many governing and regulatory competences (Weinshall, 2017), and the range of these competences varies under different models of judicial governance, depending on their distribution between judges, politicians, and other actors. Very recent political development shows that political leaders care a great deal about the regulation and control of the judiciary (Kosař & Šipulová, 2020) and that the decision to delegate these powers to judges is not taken lightly. This is, of course, to be expected, as the cumulation of delegated powers typically constrains the future choices of elected political actors in reacting to various challenges (Streeck & Mertens, 2010).

In contrast, the scholarly debate on the content and extent of judicial governance (or JSG for that matter) is still rather narrow. While many academic works engage with some elements of judicial governance, the conceptualization of its competences, structure, and actors remains rather vague. In fact, scholarship researching judicial governance is largely divorced from political science literature on the governance, its structure, and coordination between its actors (Black, 2008; Börzel & Risse, 2010; Joradana & Sancho, 2004; Mathieu et al., 2016; Mayntz, 2004)^4^ and, instead, focus selectively on the politically most salient issues: the selection, removal and disciplining of judges, and the emergence of judicial councils.

Although there are at least three dominant types of judicial governance (ministerial model, judicial council model, and court service model; for example, Castillo‐Ortiz, 2019), it is the judicial council that has been equated with a symbol of global judicial empowerment (Bobek & Kosař, 2014; Garoupa & Ginsburg, 2009; Ingram, 2012; Kosař, 2016; Parau, 2015; Parau, 2018; Pozas‐Loyo & Ríos‐Figueroa, 2011; Preshova et al., 2017; Solomon, 2018) and which dominates scholarly discussion. Judicial councils were designed as bodies insulating choices related to the careers of judges (selection, promotion, and removal) from partisan policies (Thomas, 1997) and securing adequate levels of judicial accountability (mostly via disciplining tools; Benvenuti & Paris, 2018; Vauchez, 2018). They were believed to stand in a fragile equilibrium between judges governing themselves and courts being under the complete control of political actors.

The connection between the insulation of choices related to judges' careers and judicial independence is rather straightforward. The argument that to secure judicial independence the judiciary must be institutionally insulated from other actors has not only been present in the scholarly literature (e.g., Larkins, 1996; Rios‐Figueroa, 2006, p. 4; Shetreet, 2011, p. 3), but can also be found in several documents backed by democracy and rule of law promoters such as Recommendation CM/Rec (2010) 12 on the independence, efficiency, and responsibilities of Judges of the Committee of Ministers issued by the Council of Europe, the European Charter on the statute for judges approved in 1998 by the European Association of Judges and the Universal Charter of the Judge adopted in 1999 by the International Association of Judges. However, several scholarly works suggest that structural insulation is not sufficient to secure *de facto* independence, whether measured at the level of actual decisionmaking regarding judicial careers (Spáč, 2020; Voigt et al., 2015) or at that of adjudication (Popova, 2012). Other empirical research shows that the establishment of judicial councils was also frequently tied in with expectations of better performance in terms of efficiency or quality of decision‐making, believing judges to be better equipped to regulate their own field (Urbániková & Šipulová, 2018).

In 2009, Garoupa and Ginsburg documented over 60 countries that had established judicial councils since the 1980s (Garoupa & Ginsburg, 2009). This institutional boom, together with the normative expectations laid on judicial councils, led many scholars to focus exclusively on judicial councils and derive the content of JSG, that is, the role played by judges in judicial governance, from the competences of judicial councils juxtaposed with the opposite extreme, the ministerial model. Similarly, the analysis of outputs of institutional models was dominated by a comparison of designs dominated by ministries of justice versus judicial councils (which however vary significantly in their composition, organization, and scope of competences), ignoring other institutional models which might also delegate substantive amounts of power to judges. The imprecision of such an approach was reflected in fragmented typologies.

The first typology of judicial governance was introduced in 2003, distinguishing between the Southern European and Northern Managerial models of judicial councils (Voermans & Albers, 2003). The Southern type was modeled on the Italian Consiglio Superiore della Magistratura, the archetype of modern judicial councils which was established after World War Two, and aimed at the complete insulation of the judiciary from political influence (Thomas, 1997). It delegated decisionmaking on the promotion, tenure, and removal of judges and judicial salaries to a judicial council consisting of a majority of judges and a minority of nonjudges. The model spread to Latin America and CEE. The Northern Managerial model, typical of Nordic countries, consisted of judicial councils endowed with mostly smaller managerial competences concerning court budgets and other administrative tasks, often also based on the equal participation of judges and nonjudges.

The binary typology of Southern and Northern models was later rejected by Garoupa and Ginsburg (2009), who developed a simple ordinal index of powers held by judicial councils and organized them into three groups based on two axes: the composition of a judicial council (dominated by high court judges, lower court judges, or nonjudges), and its competences, which they divided into (1) extensive (disciplining, removal, promotion, and appointment of judges), (2) intermediate (appointment of judges), and (3) minimal (housekeeping functions). In 2014, Bobek and Kosař went further and offered a new classification, which looked at governance models more generally and distinguished between five forms of judicial governance in Europe based on the character and competences of the judicial governance body: the Ministry of Justice model, the judicial council model, the courts service model, a hybrid model, and the socialist model (Bobek & Kosař, 2014). Castillo–Ortiz later used this classification to categorize judicial governance systems in 29 European liberal democracies (Castillo‐Ortiz, 2019).

In 2018, Kosař attempted to consolidate the previous piece‐meal approach to studying JSG and presented an even broader understanding of judicial governance, dividing it into eight dimensions: regulatory, personal, administrative, financial (budgetary), educational, digital, informational, and ethical. Moreover, Kosař also widened the category of actors engaging in governance of courts to include not only judicial councils and ministries, but also judicial associations, judicial academies, and court presidents. This significantly broadened the concept of judicial governance so as to cover any interaction by any actor engaged in the governance of courts, and brought the judicial governance research closer to more general scholarship on structural aspects of governance (Camacho & Glicksman, 2021; Jacobs, 2019; Mathieu et al., 2016; Shah, 2015). The evidence of more JSG actors also corresponds to trends in the global proliferation of governance agencies (Jordana et al., 2018) or agencification (Lurie et al., 2019). It shows that the many existing models of judicial governance are, in fact, much more complicated and cannot be evaluated as an exclusive dispersion of power between the executive and judicial power represented by the ministry of justice and the judicial council (Mathieu et al., 2016).

The comparison of how these models and respective typologies translate in the courts' empowerment is, however, largely undocumented. The field of judicial governance has, so far, ignored the question of how the institutional design and interaction between public institutions with delegated authority affects the regulatory output (similar reservations on institutional models and governance have recently been raised by e.g., Camacho & Glicksman, 2021; Mathieu et al., 2016, p. 265).

We have little to no empirical evidence of how much power judges hold under different models of judicial governance. Attempts at measuring the engagement of judges in judicial governance are few and limited in scope. Smithey and Ishiyama (2002), for example, presented the index of judicial power based on the analysis of constitutions. The index is, however, too narrow. It is limited to constitutional courts, and targets only the personal dimension and indicators related to the number of actors in the judicial appointment process, the degree of difficulty in removing them, and the setting of procedural rules.

The European Network of Councils for the Judiciary (ENCJ) developed a questionnaire aimed at analyzing whether judges have decisive influence in areas covering a wide set of judicial governance competences. However, the choice of competences was, again, arbitrary and does not include, for example, an educational or an ethical dimension.^5^ Even more importantly, the questionnaire merely captures *perceptions* of country experts (mostly judges)^6^ and reports governance‐related indicators as mixed with other variables. The EU Justice Scoreboard, a detailed dataset describing the performance of EU judiciaries, also does not allow one to measure the participation of judges in judicial governance, as it analyzes different parameters and changes the scale for their evaluation in individual editions (from binary yes/no to multiple choice; Hayo & Voigt, 2014, 2016).^7^ Some indicators related to judicial governance appear in the *de jure* judicial independence index suggested by Hayo and Voigt and in the judicial independence index by Feld and Voigt (2003; also Voigt et al., 2015). However, both indexes collect only limited data on power and competences held by judges and focus on measuring their independence instead. Beyond Europe, datasets allowing the measurement of the participation of judges in judicial governance are even scarcer and less complete.

In other words, although legal scholarship and international organizations, in particular, generate many hypotheses and presumptions on the positive effect of judicial empowerment on judicial independence (see e.g., the European Network of Councils for the Judiciary,^8^ the Consultative Council of European Judges,^9^ the *European Commission for Democracy through Law*,^10^ the Council of Europe^11^ and the International Association of Judges^12^), such claims are, in fact, unsubstantiated by hard data. So far, there has been no empirical research documenting the amount of power held by judges in individual models of judicial governance, nor any empirical test of the frequent assumption that the number of powers held by judges correlates positively with the output of judicial governance (be it judicial independence, efficiency, or the quality of decisionmaking). This is an apparent gap in judicial governance scholarship, particularly, considering the literature which suggests that allocation of inter‐governmental authority is one of the factors influencing the fate of regulatory and governance mechanisms (Camacho & Glicksman, 2021).

To fill this gap our article proceeds with a reconceptualization of judicial governance, based on which it presents the JSG Index. The index is complex: it traces the distribution of power on different dimensions of judicial governance and the coordination of the allocation of competences between different actors. It is also an important addition to a more general debate on the agencification and increase of regulatory and governance actors (Black, 2008; Joradana & Sancho, 2004; Mathieu et al., 2016).

This is a first step on the long road to the measurement of the power of judges in the sphere of judicial governance, which will in the future allow us to explore the causal inferences between the empowerment of judges (i.e., self‐governance) and the output of governance, the performance of the judiciary measured by various indexes of judicial independence, accountability, and efficiency.

## 3 Reconceptualizing judicial governance

Drawing on previous scholarly works (Börzel & Risse, 2010; Castillo‐Ortiz, 2019; Kosař, 2018), we adopt a broad understanding of judicial governance as a structured model of social coordination that produces and implements a set of institutions, rules, and practices which are collectively binding and which regulate how the judicial branch exercises its functions. JSG then captures the extent to which judges and courts participate in judicial governance.

The structure of judicial governance includes a constellation of actors and institutions, typically, represented by state bodies, judges, lawyers, politicians, and what we call JSG bodies: institutions established to share in judicial governance competences, including at least one judge. These are typically judicial councils, court services, judicial appointment commissions, court presidents, judicial associations, and judicial academies.

This broad delineation of judicial governance allows us to implement the most inclusive approach and capture competences in various dimensions of judicial governance. Building on Kosař's work (2018), we identified 60 competences related to eight dimensions of judicial governance: regulatory, personal, administrative, financial, educational, informational, digital, and ethical (Table 1). The list of competences was derived from the existing literature on judicial governance, judicial independence and effectiveness, and regulation of governance, as well as our own research. Some elements of regulatory dimension were already mentioned in Smithey and Ishiyama's Index (2002); Hayo and Voigt indexed the selection, nomination, approval, and dismissal of judges (Hayo & Voigt, 2014, 2016). Gutmann and Voigt (2018) correlated the transfer of judges and case allocation. Feld and Voigt's Judicial Independence Index combining *de jure* (based on legal documents) and *de facto* (based on observations of experts) measures them and then hypothesizes that if judges (or other jurists) are to appoint new judges and if the chief justice can decide about the allocation of cases, *de jure* judicial independence is greater. Feld and Voigt also include some competences related to the informational dimension as they hypothesize that judicial independence decreases if courts are not obliged to publish their decisions (Feld & Voigt, 2003).

**TABLE 1:** Dimensions of judicial governance

I. Regulatory	Competences related to establishment, abolition, or changes in the jurisdiction and procedural rules of a court
II. Administrative	Composition of a court (setting the number of judges, panels and their composition), work schedules, case assignment
III. Personal	Selection and (re)appointment of judges, promotions, removals and transfers of judges (permanent and temporary), disciplining of judges, civil and criminal prosecution, evaluations of judges
IV. Financial	Size of a court's budget, salaries of judges
V. Educational	Compulsory education (plan and structure) and further training and education of judges
VI. Informational	Publication of rulings, recordings of trials, annual reports, case assignment, disclosure of judges' property, political affiliation and some personal information
VII. Digital	Placement of servers with online data
VIII. Ethical	Preparation and interpretation of the code of conduct, off‐bench activities of judges, communication with media

*Source*: The authors. For a complete list and coding rules, see Appendix.

Van Dijk and Vos (2018) mention budgetary arrangements, the determination of judges' salaries, promotions, evaluations, and management of the courts' tasks. The informational dimension appeared in Feld and Voigt's Judicial Independence Index. We also took inspiration from various measurements of the *de facto* independence and governance of public broadcasters (Hanretty, 2009; Juwayeyi, 2017) and the independence of central banks (especially concurrence of functions, Cukierman, 1992), as well as from more general conceptualizations of public regulatory agencies' competences (Jordana et al., 2018).

- The regulatory dimension (five competences) covers all decisions on the establishment, abolition, merger, division, and jurisdiction of courts. These competences are typically regulated by the legislative power (parliament), but we are interested in whether judges take any part in the subsequent adoption of changes to these regulatory competences. The ability to comment on legislation related to courts and judges is one of the most typical results of judicial empowerment and offers judges an important voice in the legislative process.
- The administrative dimension (nine competences) targets administrative decisions on the courts' functioning, such as the overall number of judges assigned to a given court, the structure (number and composition) of panels, the overall number of assigned administrative personnel, and law clerks, but also questions of case assignment and reassignment (sometimes referred to as case allocation) and the evaluation of courts (in terms of the quality of their decision‐making, finances, budget, etc.).
- The personal dimension (19 competences) groups together all decisions on the career and accountability of a judge. It covers the broadest range of competences from selection and appointment, transfer, and removal (including temporary), and disciplining of judges to judicial performance evaluation, financial bonuses, and the criminal liability of judges.
- The financial dimension (four competences) addresses decisions on the size and allocation of a court's budget and the salaries of judges.
- The educational dimension (five competences) deals with decisions on the compulsory and further education of judges and the training of judicial candidates, asking who decides and creates the structure and content of education, including the certification of a judge who can then apply for a vacant position.
- The informational dimension (nine competences) covers competence to determine which judgments are published, to decide on recordings of trials, the obligation to disclose judges' property and their political affiliation, statistics related to caseload, annual reports on individual courts and on the whole of the judiciary.
- The digital dimension (one competence) relates to the physical placement of servers, asking whether judges, or the executive manage the online servers. This seemingly technical question gained in importance with the digitalization of justice, as the ability of courts to manage their own clouds and servers allows them great flexibility in terms of internal management (introduction of new search engines, case management systems, etc.) and transparency towards the public.
- The ethical dimension (eight competences) captures decisions on the preparation and interpretation of the code of judicial conduct, communication by judges with media, and the regulation of their off‐bench activities. It is worth mentioning that the list of ethical competences includes several which might also be covered by the ethical code (listed as an individual ethical competence). We decided to list the most important of those separately to cover information on jurisdictions which do not enact formal ethical codes.

This eight‐dimensional conceptualization of judicial governance allows us to explore the extent of JSG and its correlation with judicial independence, accountability, transparency, and effectiveness, both as a whole as well as in individual areas, which remain so far under‐researched.

## 4 Constructing the JSG Index

The multidimensional conceptualization of judicial governance carries with it vast comparative potential as it allows us to compare the *de jure* power held by judges in countries with different models of judicial governance and hence look beyond institutions that play a central role in different countries—be they ministries of justice, judicial councils, or court service systems. Instead, the JSG Index shows the extent to which judges participate in the regulation and execution of competences related to judicial governance.

After the identification of 60 indicators in the field of judicial governance, the construction of the JSG Index followed in three stages: the construction of the coding scale, the collection and coding of primary data (Section 4.1) and data aggregation (Section 4.2).

The multidimensional index has two important characteristics related to its applicability. First, we designed and tested the JSG Index for general domestic courts (i.e., civil, criminal, and administrative) in constitutional democracies. We expect that the index will be easily transferable to supreme courts, special tribunals, constitutional courts, and international courts, after a little tweaking to reflect specific competences tied to a court level, for example, the promotion of a supreme or constitutional judge would have to be coded as not available, and so on. The JSG index is therefore primarily applicable to domestic lower courts with general jurisdiction. In this sense, it is unique, as most of the political science indexes introduced in Section 2 concentrated only on constitutional or apex courts and did not take lower courts into account.

Second, our index reports the scores of *formal* delegation of judicial governance competences. In doing so, it captures only the *de jure* assignment of competences—the JSG Index uses the analysis of formal legislation (i.e., constitutions, statutes, sub‐statutory legal acts, and the relevant case law) to calculate the scores.^13^ While it is true that informal institutions and practices might have significant influence over the division of powers among individual actors of judicial governance, we consider the *de jure* mapping an important step for the future analysis of outputs of different institutional models of judicial governance as well as for other research analyzing how decision‐makers construct various regulatory governance designs (Gilardi, 2008; Hanretty & Koop, 2012; Jordana et al., 2011; Koop & Lodge, 2014; Mathieu et al., 2016). Here, we also draw inspiration from different research on multi‐level governance systems suggesting that formal decision‐making structures are critical for inducing cooperation between regulatory actors (Koop & Lodge, 2014, p. 1314; Mathieu et al., 2016, p. 253). Moreover, we have discovered that differences in the formal regulation of competences run not only across countries, but also across individual competences. As we demonstrate in the empirical section, the level of formal regulation changes in time in every JSG model, and spurs the need for further theoretical work on a differentiation between individual dimensions of judicial governance. We explain the variances in this variable later on. The existing variance nevertheless suggests that when analyzing the output of judicial governance and its regulation, the informal influence of individual actors should be studied together with formal arrangements and institutional setups, to understand the role of formal structure and power distribution on the *de facto*, informal empowerment of individual judicial governance actors.

In what follows, we explain the construction of the JSG Index and its scale, address the core methodological choices related to its creation, and elaborate on the method of evaluation of the role of judges among other JSG actors.

### 4.1 Coding scale and data collection

One of the core challenges of the index's construction was the development of a coding scale. All competences included in the index can be held by judges (sitting at courts, disciplinary panels, judicial councils, etc.), or nonjudges (e.g., politicians, most often the Minister of Justice or the President, MPs, public servants, civil society, and legal scholars; for more on actors see Appendix, part I. C).

In order to assess the extent of judicial involvement in specific JSG competences, we took inspiration from other composite indexes (Gutmann and Voigt's scale used in *de jure* independence indicators (2018)) and created a scale capturing the interaction between judges and nonjudges.

The JSG Index therefore codes individual competences on a five‐point scale ranging from 0 to 1. We designed the scale bearing in mind the argument raised by Hanretty and Koop (2012) that ordinal response categories must be either intuitive or well justified, and we opted for the intuitive approach. The first step in assessing the involvement of judges was to identify all actors in whom the law vests the competence in question. For every competence, we were then interested in whether judges (a) decide on the competence on their own without the involvement of other actors, (b) decide independently next to a different actor (e.g., the disciplinary proceedings against a judge can typically be initiated independently either by the court president or by the Minister of Justice), (c) have to negotiate with nonjudges, as both actors have the power of veto, (d) must be obligatorily consulted,^14^ or (e) are not involved at all (Table 2). The scores were spread equally among five categories. We are aware that individual judicial governance actors might perceive the impact of their involvement differently; however, given the lack of understanding of how far individual categories of influence are from each other, we considered the equal distribution as the most precise and objective approach (for a justification of a similar approach see Mathieu et al., 2016).

**TABLE 2:** A scale for Judicial Self‐Governance (JSG) Index scoring

To what extent are judges de jure involved in deciding on a competence?	Value
Judges decide	1
Judges decide and nonjudges decide (independently of each other)	0.75
Judges negotiate with nonjudges (both have veto power)	0.5
Judges are consulted	0.25
Not at all	0

*Source*: The authors. For more details on the coding and design of the scale see the Appendix.

We also had to take into consideration that the executive power frequently assigns a particular competence to a variety of JSG bodies (such as a court service, a judicial council, a selection committee, a disciplinary panel: see Kosař, 2018), which may also be composed of both judges and nonjudges. For each JSG body, we first inquired whether judges hold a decisive majority in it or have to decide together with their nonjudicial peers. We therefore coded competences that were delegated to JSG bodies on the basis of the requirement that judges are involved in adopting a decision. For example, if the judicial council consists of 11 members, six judges and five nonjudges, and adopts decisions with a two‐thirds majority, we would count the judicial share of the decisionmaking of this council as 0.5 (as judges need to negotiate with their nonjudicial peers).

The last remark relates to what we call *derived de jure* regulation of competences. Not all competences are necessarily explicitly regulated by the law. In some cases, the law does not regulate the delegation of the competence to any actor and the authority of an actor is derived from other provisions or competences of greater power. In such cases, we implement the logic of the regulatory independence of JSG actors and assign the unregulated competence a default value. This default value captures who can exercise the competence in the absence of its regulation, that is, it reflects the degree of autonomy formally left to the actor. For example, if the law does not regulate case assignment, we code the competence as a 1 (the closest interpretation would be that the legislation did not bind the courts to a particular form of case assignment and left the competence to their discretion). In a different scenario, if the law does not regulate the temporary assignment of a judge to a nonjudicial body (typically an internship at the Ministry of Justice), but any other transfer of a judge is a competence of the Minister of Justice, we code the competence as a 0 (the closest interpretation would be that such a competence was not given to judges and it either is not exercisable or requires explicit authorization, as the assignment of judges in general is a competence of the Minister of Justice). We manually coded and verified all default values in every jurisdiction.

### 4.2 Process of aggregation

As mentioned in the previous section, we understand judicial governance as a multidimensional compensatory index where a high level of power in one dimension can substitute for a low level of power in others. To put it differently, there is no hierarchical order between individual dimensions, and at this point, all are considered equally important for the concept. Considering the compensatory nature of the JSG Index and unequal distribution of competences between dimensions, we opted for a simple calculation of the JSG score, dividing the aggregate score in all competences by the number of competences. In an ideal scenario, we would have aggregated the scores in each dimension and then calculated the JSG score as an average of the scores in individual dimensions. However, this approach would work properly only with a roughly equal distribution of competences per dimension, which is not the case with the JSG Index. Incidentally, the dimensions typically identified as important for judicial governance (personal, administrative, and financial) have more competences than others, automatically allowing the JSG Index to give these competences more weight in the overall score.

Alternatively, either we could have approached judicial governance as a noncompensatory concept where some dimensions (e.g., personal) would be considered more important for the overall score and given different weight, or we could have searched for a more parsimonious arrangement of dimensions in the concept, but this can be re‐evaluated only with a sufficiently large dataset. Although we are aware that individual competences may have different importance in the regulatory field (Hanretty & Koop, 2012), we opted for equal weights assigned to all judicial governance competences. This important methodological choice was influenced by an absence of existing theoretical or empirical data on the relative importance of some of the judicial governance dimensions introduced only by very recent scholarship (Kosař, 2018). Moreover, considering the unequal distribution of competences in individual dimensions of the JSG Index, we found the simplest method of aggregation with equal weights assigned to all competences to be the most reasonable and least arbitrary choice, which can be improved in future by more empirical research or perceptions of JSG actors on the importance of individual dimensions (for a similar approach see Gilardi, 2008, p. 880; Hanretty & Koop, 2012, pp. 204–205; Mathieu et al., 2016, p. 261). A more detailed discussion and testing of various methods of aggregation is included in Appendix 2.

### 4.3 Theoretical dilemmas

The design of the coding scheme presented us with three substantive challenges related to the evaluation of actors holding individual judicial governance competences: (1) definition of a “judge,” (2) the agent/trustee dilemma of selected JSG members, and (3) the simultaneous execution of judicial and nonjudicial roles by some JSG members. Consequently, both challenges instigated important methodological decisions which influence the scoring implemented in the index.

First, the *distinction between judges and nonjudges* is obvious in most cases, but there are some borderline examples such as part‐time judges, lay judges or prosecutors in Italy and France. For the purposes of our JSG Index, “judge” means a *full time professional* judge. To determine who is a full time professional *judge* we use the rule of dependency and look at who can, in a given jurisdiction, (*de jure*) issue instructions on how a given actor (such as a prosecutor or general secretary) should act substantively. If this actor decides independently, we count her as a “judge.” In contrast, if the executive power may issue instructions on how this actor should act in substance, we count her as “nonjudge” (for more details, see Appendix, part I. C). For this reason, we coded Italian prosecutors as judges (because they are both *de jure* and *de facto* understood as members of the judiciary), while we would code French prosecutors as nonjudges (even though they are formally defined as a part of the judiciary but *de facto* act differently). A “nonjudge” is then defined as anyone who is not a “judge,” even though we are aware that there are a variety of “nonjudges” involved in judicial governance (politicians, members of other legal professions, civil society, and experts such as human resource specialists or economists) and that each group of these “nonjudges” can raise specific problems.^15^

Second, *the agent/trustee dilemma* targets the composition of JSG bodies in general and judicial councils in particular. As indicated in Section 2, most scholarly works differentiate between judicial councils based on the participation of judges in their composition (councils with judges in the majority, councils built on parity between judges and nonjudges, and councils with a majority of nonjudges). Nevertheless, the logic behind the composition of judicial councils differs. Whereas in some judicial councils judges have to comprise the majority of their members, in others, the composition criterion merely states that the majority of the judicial council's members have to be selected by judges, and it is not clear whether judges can select only judges or also nonjudges and vice versa. For instance, the Italian Constitution does not lay down how many judicial members have to sit on the council, only that judges shall select the majority of them. Similarly in Slovakia, the Constitution and the Law on the Judicial Council posit that judges shall elect nine members, whereas parliament, government, and the President appoint three members each, without specifying which pool of candidates each nominator must choose from. As a result, the Slovak President, parliament and government often elected judges to the Slovak judicial council. This significantly shifts the balance of power envisaged by the Slovak Constitution (the parity system) as judges then become a majority on the judicial council (Spáč et al., 2018).

This led us to a dilemma whether to score the competences assigned to JSG bodies with mixed composition on the basis of the number of judges in the body (composition principle) or on the influence of judges on the selection of the body's members (selection principle). The existing scholarship identifies two distinct approaches to this dilemma. First, the agent model argues that the act of delegation of a power (or a competence) defines the relationship between the principals and the agents to whom they delegate the power. In the domestic courts setting following the agent model, judges are given the competence to enforce the choices of constitution‐makers against recalcitrant legislative majorities (Dyevre, 2015). Second, the trustee model casts domestic judges as trustees of the political system. Principals choose to delegate the powers to trustees to harness the authority of the trustee and enhance the legitimacy of the decisionmaking process (Alter, 2008). Trustees are not apolitical or immune, but they are less manipulable via recontracting mechanisms, and hence, less dependent on a principal's political will (Dyevre, 2015).

Translating the agent–trustee dilemma into the process of selection of members of JSG bodies, the agent model presupposes a relationship of dependency between the nominators (judges or nonjudges) and the members (again judges or nonjudges) it selects, while the trustee model sees the JSG bodies' members as more independent. While acknowledging that behavioral patterns of members of JSG bodies require deeper theorizing, for the purposes of the JSG Index, we consider the trustee framework a better theoretical point to build on. As principals in judicial governance typically cannot dismiss members of JSG bodies they have selected of their own free will, there is no formal dependency.^16^ Neither judges nor political actors can, formally, instruct the JSG bodies' members on how to act and how to execute their role inside the JSG body. Therefore, we expect judges in principle to act as representatives of the judiciary, irrespective of their selection method.^17^

The third methodological dilemma arising in some jurisdiction is what we call *judicial nesting*. Some jurisdictions entrust JSG competences to bodies or actors who cannot easily be identified as a part of the judiciary, the executive or the legislature or as agencies (Lurie et al., 2019). A typical example would be general secretaries, registrars, and directors of judicial offices^18^ (Spáč et al., 2018) or the German practice of temporary assignment of judges to the Ministry of Justice. In all these situations, we again use the rule of dependency and look at who can (*de jure*) issue instructions on how the body should act substantively. If the given body is controlled by judges and decides independently of the political branches, we count it as “judges.” If the executive power or another external actor may issue instructions on how this body should act in substance, we count it as “nonjudges.” For this reason, we coded German judges assigned to the Ministry of Justice as “nonjudges” because these judges do not have the agency to decide independently of the Ministry of Justice and must *de iure* follow the Ministry's instructions.

## 5 Index validation: Empowerment of judges in different models of judicial governance

The purpose of this section is to validate the JSG Index by assessing whether the operationalization of the concept and our measurement reasonably reflect the concept we intend to measure. As said by Adcock and Collier, “valid measurement is achieved when scores … meaningfully capture the ideas contained in the corresponding concept” (Adcock & Collier, 2001, p. 530). We do so on the longitudinal data for Czechia, Slovakia, Italy, and Germany. As regards, the measurement error and reliability of our measurement, we aim for random error which may be pertinent to the fact that all data were hand‐coded^19^ based on five sources: constitution and constitutional laws, organic laws, ordinary statutes, sub‐statutory legal acts, and case law. For all four countries, we coded at six points in time: the years 1994, 1999, 2004, 2009, 2014, and 2019.^20^ The chosen timeframe allows us to control changes in the power held by judges compared with the most important historical and political events (such as the transition from the communist regime or the accession of CEE countries to the EU). Focusing on legal documents and powers held by judges *de jure*, we avoid the problem of contextual specificity of measurement validity as the approach undertaken can be reasonably expected to be valid across contexts.

We purposely selected two pairs of countries, which allowed us to observe the development of JSG on two axes: old versus new members of the EU, and ministerial versus strong judicial council models of judicial governance. As previously explained, the Italian judicial council (Consiglio superior della magistratura, “CSM”) is among the oldest in the world. It was established in 1958 and originally focused almost exclusively on the insulation of judges from political influence via the regulation of judicial selection, the allocation, transfer, and promotion of judges, the initiation of disciplinary proceedings and, to a small extent, also budgetary questions (Benvenuti & Paris, 2018). Until 2006, the CSM also executed competences concerning education and training, which were later transferred to the High School of the Judiciary. The CSM is presided over by the President of the Republic. It consists of 27 members,^21^ approximately two‐thirds of whom are judges (including the First President of the Supreme Court *ex officio*). Although it was used as a model for new EU member states (Bobek & Kosař, 2014), the scholarly assessment of the CSM and its role in the Italian judiciary is mixed at best. While the CSM successfully prevented the capture of the Italian judiciary in the Berlusconi era and ensured the structural independence of the judiciary (Dallara, 2015), the CSM and the Italian judiciary in general have been marred by many corruption scandals (Benvenuti & Paris, 2018) and failed to avoid politicization via *correnti* within the CSM and judicial associations (Guarnieri, 2004; Sallusti & Palamara, 2021).

Slovakia introduced the judicial council (Súdna rada Slovenskej republiky, “SRSR”) in 2002. The establishment of the SRSR closely correlated with accession to the EU, as well as transition from the semi‐authoritarian regime of the first Slovakian prime minister, Mečiar, whose government managed to foster a strong corruption network within the judiciary (Láštič & Spáč, 2018). Based on the EU and CoE recommendations, Slovakia modeled its judicial council to a large extent on the Italian one. The SRSR consists of 18 members, nine elected by judges and nine by political branches (the Parliament, President, and the government each elect three members). In theory, the SRSR aimed for parity in its composition, but many Slovak political actors in fact appoint judges as well. Thus, the SRSR is overwhelmingly a judicial body by its composition. Like the Italian CSM, it is a strong type of judicial council with significant competences in the selection, promotion, and disciplining of judges, but it also has influence on court organization, work schedules, and ethics regulation (Kosař, 2016). The existing scholarship argues that the Slovakian judicial council failed in promoting judicial independence and, instead, strengthened the pre‐existing informal corruption networks and further isolated the judiciary (Kosař, 2016; Spáč, 2020; Spáč et al., 2018).

Both in Slovakia and Italy, judicial councils are the most important, but not the only, actors of judicial governance. A substantial role is also played by decentralized judicial boards (they evaluate judges for purposes of promotion to Italian appeal courts, and have various consultative powers at all court levels in Slovakia), judicial associations, which have a substantive influence on the selection of judicial councils' members, judicial academies and court presidents. Furthermore, some competences are also retained by ministries of justice, which manage the material resources of courts, supervisory powers (in case of Italy), finances (in case of Slovakia), or the day‐to‐day administration of courts (Benvenuti & Paris, 2018; DiFederico, 2012; Spáč et al., 2018).

In contrast, Czechia is often labeled the black sheep among the CEE countries (Bobek, 2008; Kosař, 2018; Wittreck, 2018). Although the introduction of a judicial council has been on the table since 1998, despite considerable pressure from the apex courts and the judicial association (Beers, 2012) it has not been implemented. Past legislative proposals lacked wider political support and Czechia instead stuck to a ministerial model, coupled with a very strong role for court presidents, who informally execute many competences because they were informally delegated to them by the Ministry of Justice. The central body of judicial governance; however, remains the Ministry of Justice, with substantial personal, organizational, and financial powers. Next to the Minister of Justice, court presidents share the competence to initiate disciplinary proceedings. They also decide on appointments, secondments, promotions and transfers of judges, control work schedules, and, informally, have hence had a certain indirect influence on case assignment. Court presidents are also *de facto* gatekeepers of the Czech judiciary, as they, instead of the executive, often pick new judges (Blisa et al., 2018; Kosař, 2017: p. 100).

Germany is then an example of an “old” EU country that has never seriously even considered the establishment of a judicial council. Judicial governance is dominated by the federal Ministry of Justice which administers federal courts, while state ministries oversee courts at the state level. Their competences include recruitment, training, examinations, as well as issues related to the administration of buildings, equipment, and staff (Seibert‐Fohr, 2012, p. 456). However, as with Czechia, there are strong elements of self‐governance represented by influential court presidents (disciplining, transfers), judicial boards (case assignment) and councils of judges (employee participation; Wittreck, 2018). Arguably, despite the failure to implement a judicial council, both Germany and Czechia score more highly in indicators describing the quality of the rule of law, judicial independence and judicial accountability than Italy and Slovakia (e.g., Staton et al., 2019).^22^

**FIGURE 1:**
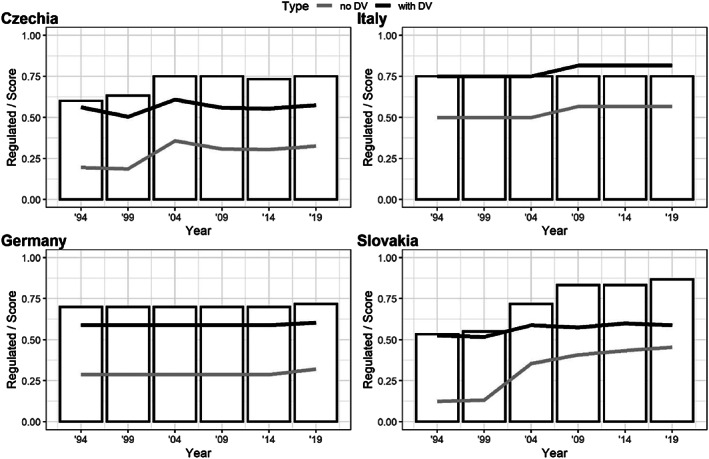
Judicial Self‐Governance (JSG) Index scores 1994–2019, with and without default values, contrasted with the level of judicial governance regulation. *Source*: The authors.

Following the scholarship and theories presented in Section 2, we posit three research questions which are then explored using the results of the JSG Index plotted in Figure 1:

(1) Can we observe a general trend of judicial empowerment in all institutional models of judicial governance?

The dominant literature on courts and judicial governance suggests the overall trend of judicial empowerment (Bunjevac, 2020; Kosař, 2016; Piana, 2010) following the increasing importance of courts and the judicialization of politics (Ferejohn, 2002; Hirschl, 2004; Tate & Vallinder, 1995). Although early scholarship connected empowerment almost exclusively with the establishment of judicial councils (Albrecht & Thomas, 2009; Autheman & Elena, 2004; Bobek & Kosař, 2014; Garoupa & Ginsburg, 2009; Parau, 2015; Piana, 2010; Pozas‐Loyo & Ríos‐Figueroa, 2011), many researchers observed an increasing agencification of other actors of judicial governance, demonstrated mostly in the growing role of court presidents (Blisa et al., 2018; Spáč & Kosař 2021), court directors (Lurie et al., 2019) or judicial associations (Beers, 2012). Following these works reporting the increasing importance of numerous JSG actors, we ask whether there is a general trend of judicial empowerment observable across jurisdictions with different models of judicial governance.

(2) Does the JSG Index report significantly different scores of judges' involvement in countries with different models of judicial governance? Are there substantive differences between countries of Western and Eastern Europe?

Building on the first question, we are inquiring whether there is a considerable gap between the ministerial and the judicial council models of judicial governance, as well as how different categories of judicial councils (Bobek & Kosař, 2014; Castillo‐Ortiz, 2019; Garoupa & Ginsburg, 2009) fare in the JSG Index. Moreover, we are also examining differences based on the geo‐political location of judicial governance systems. The Eastern EU enlargement put tremendous pressure on CEE countries in terms of institution building. Judicial councils became one of the blueprints recommended by many international organizations (Piana, 2010). Their implementation was not uniformly successful though (Kosař, 2016; Spáč et al., 2018). In a paired comparison of four countries, we are interested to see if there are any differences in powers held by judges in the ministerial versus judicial council models in old and new, post‐communist EU member states.

(3) If there is a trend of judicial empowerment, is it associated with increasing regulation?

The third question reflects on a fairly new branch of scholarship pointing to processes of agencification (Lurie et al., 2019; Verhoest, 2013) and growing informal competences of JSG actors (Juwayeyi, 2017; Tsereteli, 2020). The complexity of the field of judicial governance is increasing in time. New regulatory trends and emerging agencies give rise to completely new competences (such as digitalization, online publication of data, preparation of ethical codes, etc.). So far, we have not had much empirical information on what is happening with these new competences: how regulated the field really is, and how the new competences are distributed among individual judicial governance actors. We therefore ask whether the rise of judicial powers (if there are any) is associated with an increasing regulation of judicial governance. Are new judicial governance competences allocated to judges? Or do judges mostly take over already regulated competences?

### 5.1 How differently do models of judicial governance score in the JSG Index?

The JSG Index scores of Czechia, Germany, Italy, and Slovakia, plotted in Figure 1, tell us three different but equally compelling stories: the scores of self‐governance in explicitly regulated competences, that is, without default values (Figure 1, grey lines); the score of self‐governance after computing default values in competences which are not explicitly regulated but derived from other competences (Figure 1, black lines); and the level of regulation (Figure 1, bars) informing us how regulated all competences are.

At first sight, the development of JSG Index scores without default values (grey lines) corresponds with a narrative that equates judicial councils with judicial empowerment. While we can observe a certain increase in JSG in all four cases, Italy clearly outpaces the other models with the highest score—Italian judges control more than half (56.7%) of all judicial governance competences. On the other hand, both the Czech and the German ministerial models score significantly lower (32.5% and 32%, respectively). In 1994, post‐communist Czechia and Slovakia started with a very small amount of regulated JSG (19.6% and 12.5%) but delegated a considerable number of competences to judges in the wake of the EU accession process (an increase to a little over 35% in both countries). Rising judicial empowerment is most prominent in the Slovak case, as, even after the establishment of SRSR in 2002, Slovakia apparently kept delegating new competences to judges. In 2019, the JSG Index places Slovakia in Italy's slipstream, with 45.4% of competences governed by judges and JSG bodies.

However, this story changes dramatically after the addition of default values for those competences which are not explicitly regulated but are derived from other legal provisions or case law (black lines). While Italy remains the leader (with 81.7% of competences held by judges in 2019), both the German and Czech scores demonstrate the presence of substantial self‐governance elements and, even more surprisingly, as of 2019 the German and Czech ministerial model actually empowers judges to the same extent as the Slovak model of a judge‐dominated judicial council (60.4% in Germany and 57.5% in Czechia, vs. 58.8% in Slovakia). These results confirm some critical scholarly voices arguing that the German ministerial model should not be perceived as the supreme rule of the Ministry of Justice, which would exclude judges from judicial governance (Bobek & Kosař, 2014; Seibert‐Fohr, 2012; Wittreck, 2018). When we look more closely at our data, the JSG Index captures the important role of German judges in several administrative, disciplinary, and personal issues, especially via court presidents and collective JSG bodies, namely judicial boards (*Präsidien*, which deal with the assignment and composition of judicial panels), judicial appointment councils (*Presidialräte*, which act mostly in the area of promotions) and committees for selection of judges (*Richterwahlausschüsse*; see Riedel, 2013, p. 46; Sanders & von Danwitz, 2018, p. 796). A similar observation can be made for Czechia, where the JSG Index captured the significant participation and decisive voice of judges in many judicial governance competences. This corresponds with literature stressing the dominant position of court presidents, who have decisive influence over judicial careers, disciplining and the day‐to‐day administration of the courts (Blisa et al., 2018; Kosař, 2016, 2017).

A comparison of Czechia and Slovakia sheds even more light on the relationship between the existence of a judicial council and the empowerment of judges, given that these are two countries with fairly similar political and socio‐cultural backgrounds. Consistently with the expectations of the prevailing scholarship on judicial councils, a simple count of regulated competences shows the clear dominance of JSG in Slovakia. However, after the computation of unregulated competences, the Czech model of judicial governance scores almost as highly as the Slovak. The results might suggest that while the judicial council model explicitly regulates more competences, the Czech ministerial model with strongly positioned court presidents allows the delegation of many unregulated competences to judges. This finding is particularly important in the light of studies showing that judicial councils might pose too many risks for countries with a poorly embedded rule of law and judicial independence culture, as they are prone to easy political capture (Popova, 2018, 2020; Kosař, 2016).

The story of judicial empowerment becomes even more compelling when one compares the development of JSG with the regulation of judicial governance (bars in Figure 1). First, we can observe a gradual increase in legislative regulation of judicial governance. This trend is particularly visible in two post‐communist states, Czechia and Slovakia, which entered the 1990s with a fairly low level of regulation, possibly as a legacy of the previous omnipotent influence of a communist General Prosecutor. This however changed during the EU accession process (1998–2002) when pressure from the European Commission arguably prompted domestic discussions on how to reform post‐communist judiciaries (Kosař, 2016; Spáč, 2020). The extent of regulation is highest in the personal dimension, where almost all competences in all four countries are regulated by the law. On the other hand, all four countries have the lowest levels of regulation in the digital, ethical, and informational dimensions, all fairly new ones which are only slowly attracting the attention of legislatures.

Second, a comparison of the level of regulation (bars) and scores in explicitly regulated competences (JSG score without default values, grey lines) suggests that while there is an increasing trend legislatively to regulate judicial governance, the newly regulated competences are almost always vested in the hands of judges. We can therefore argue that judicial empowerment is formalized. Moreover, for all the countries but Slovakia, the line showing an overall increase in JSG scores (with default values) copies that of regulated competences (without JSG values). This means that in Italy, Germany, and Czechia, we see the creation of new judicial governance competences, and these convey power to judges. On the other hand, Slovak regulation targeted some competences which were previously only derived as held by judges. In other words, Slovak regulation became more precise in time, explicitly delegating power to judges even in those competences which they already held. One of the possible explanations of this phenomenon was already raised by institution‐building scholarship, which suggests that countries that face problems with unhealthy informal practices also build more formal institutions (Popova, 2020).^23^ This would also explain why Slovakia, tackling a vast corruption network in the judiciary after the fall of Mečiar's semi‐authoritarian regime, has the steepest increase in judicial governance regulation, surpassing even that of Italy.These findings therefore suggest that our JSG Index reports increasing judicial empowerment, consistently with major JSG literature (Bunjevac, 2020; Garoupa & Ginsburg, 2009; Kosař, 2018; Parau, 2015; Piana, 2010), but it also demonstrates the increase in all models of judicial governance. What is more, this general trend of empowerment is typically formalized—either countries regulate new competences and delegate them to judges (Germany, Czechia, and Slovakia), or they regulate derived competences previously executed by judges and formalize their power. Furthermore, the age of the models seems to be of more relevance than the East–West division. The bigger caveat to this finding is that the absence of a judicial council does not automatically bring with it less judicial governance regulation, although we might be tempted to hypothesize that ministerial models which delegate the execution of competences to court presidents might be more ridden by informal institutions.

### 5.2 Dimensions of judicial governance

The important characteristic of our JSG Index is its compensatory character. Given the broad eight‐dimensional understanding of judicial governance we implemented in the index, we looked further into the role of individual dimensions in the evaluation and understanding of JSG. We posit that, like other political concepts, such as democracy or populism (Møller & Skaaning, 2012; Wuttke & Schimpf, 2020), JSG might also present a noncompensatory model where individual dimensions are not interchangeable and, for example, the lack of personal competences cannot be compensated for by the vast power of judges in administration, the budget, education, or ethics. To substantiate this suggestion, we compare the scores of all four countries (JSG with default values) in eight dimensions. For a more detailed explanation of the aggregation of results as well as for their comparison with other alternative approaches to aggregation see Appendix, Section 2. E.

**FIGURE 2:**
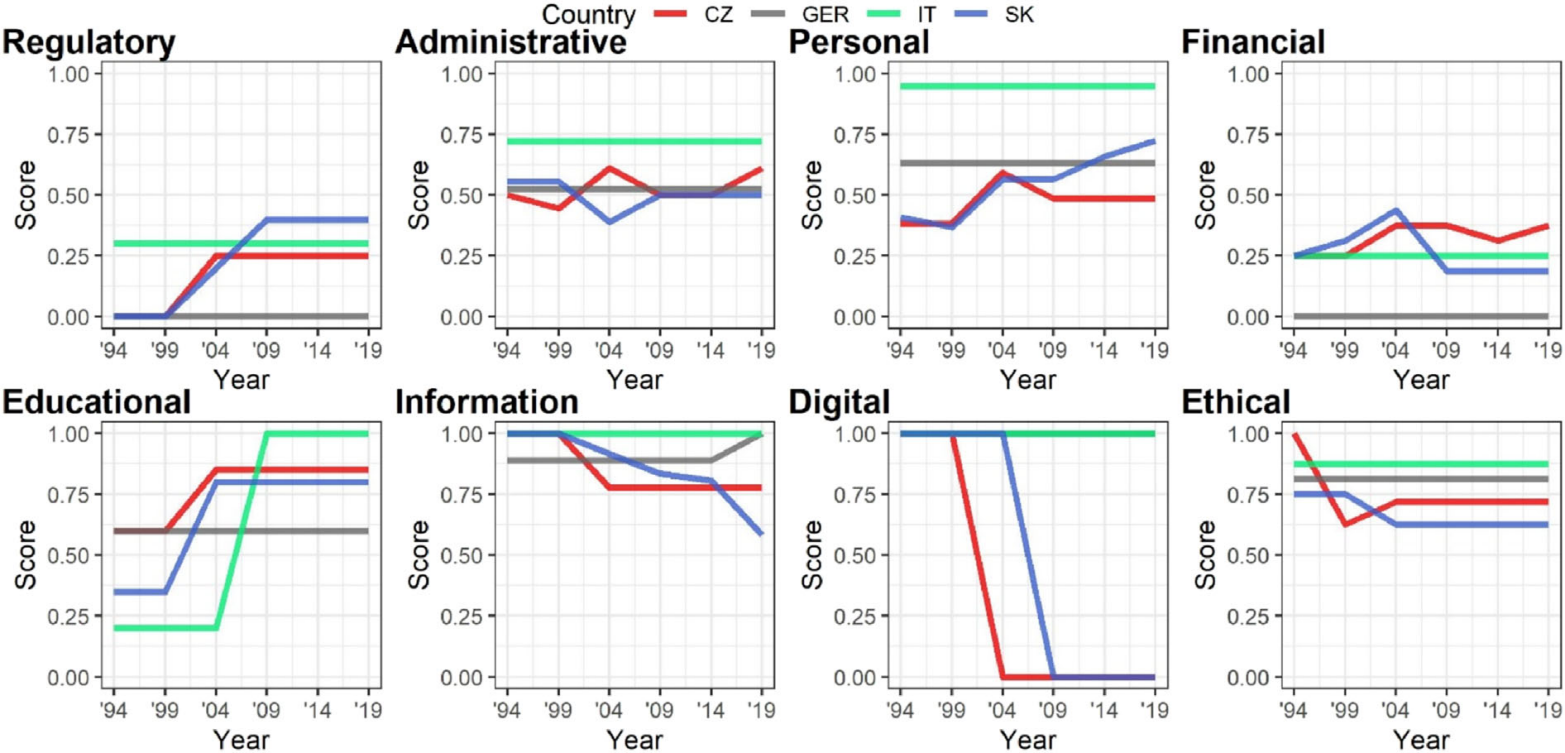
Scores for individual dimensions with default values. *Source*: The authors.

Figure 2 shows that in terms of power shared by judges and politicians, the core dimensions of judicial governance, the personal, and administrative are mostly balanced (with the exception of Italy), while the “younger” dimensions, such as the educational, informational, and ethical, are primarily vested in the hands of judges. There are some differences between the four countries, particularly in the case of Italy, where the CSM does not compensate for the influence of judges over the judicial careers of their peers with a political element. On the other hand, the Slovak SRSR model, although still categorized as strong, is more balanced and the regulation of both personal and administrative dimensions comes with many checks and balances, particularly in the selection and dismissal competences.

The steepest increase in judicial powers occurs in the educational dimension. This trend corresponds to the establishment of judicial academies and agencification of other JSG actors, like court presidents or judicial associations, which took over many competencies previously carried out by ministries of justice (Lurie et al., 2019; Wittreck, 2006). The educational dimension has so far largely escaped scholarship's focus on judicial empowerment. The few legal works on the role of judicial academies moreover suggest that the dimension is not taken as particularly politically salient (Benvenuti & Paris, 2018; Wittreck, 2006). However, the significant increase of the role of judges, combined with increasingly pressing debates on the negative effects of encapsulation, inbreeding, and judicial corporativism, suggests that the distribution of educational competences and their eventual relationship with judicial efficiency and independence should provoke more consideration (Minkner, 2015; Parau, 2018).

The split in the digital dimension between the absolute power of judges (Slovakia and Germany) and absolute control by the Ministry (Italy and Czechia) boils down again to the short life of this dimension in which we have so far identified only one competence.

The financial dimension proves to be politically salient and allows only limited participation by judges in the decisionmaking and administration of budgets. The surprising score in the financial dimension belongs to Czechia, where it is the court presidents who administer and are held accountable for courts' budget. The regulatory dimension, understandably, has the lowest engagement of judges, as we expect that establishment and legislative changes to courts' jurisdiction rests in principle in the legitimately elected parliament.^24^ From this perspective, the obligation to consult judges on legislative changes in the regulatory dimension is a significant acknowledgment of the involvement of judges in all aspects of judicial governance.

The variation in the development of individual dimensions, therefore, prompts us to theorize that the competences included in the JSG Index could be clustered and re‐evaluated to create a noncompensatory model which would assign different strengths to dimensions with different political salience. The next research step would therefore be a factor analysis of the scores of individual dimensions of judicial governance across more European countries and the subsequent analysis of whether the institutional setups of particular models of judicial governance are similar in terms of their JSG scores.

### 5.3 Conclusion and avenues for further research

This article introduces the new JSG Index, which allows us to measure the power allocated to judges in various models of judicial governance. Based on the results of the JSG Index, we argue that the differentiation between systems where courts are governed by judicial councils on the one hand and ministries of justice on the other (cf. ENCJ, 2017; Mikuli et al., 2019) does not sufficiently capture the complexity of the regulatory field and interaction between its actors.

More specifically, the application of the JSG Index to Czechia, Germany, Italy, and Slovakia shows that while a judicial council vests considerable powers in the hands of judges, the trend of judicial empowerment is present also in countries with other institutional designs. Although Italy and Slovakia, jurisdictions with a strong model of judicial council, scored more highly in the JSG Index, the differences between them and Germany and Czechia (ministerial model) are not as significant as the institutional convergence trends and positions of supranational organizations suggest (Castillo‐Ortiz, 2019; ENCJ, 2017; Mikuli et al., 2019; Parau, 2018; Van Dijk, 2021). Both Germany and Czechia actually *de jure* vest considerable powers and influence in the hands of judges (via court presidents, district judicial boards, or judicial academies).

To put it more simply in terms of our three research questions mentioned in Part 5, the JSG Index shows three trends. First, there is an increase in JSG in all models of judicial governance. Second, we can see this increase across countries with various forms of institutional regulation of judicial governance and both in the West as well as East Europe. That means that judges can actually hold many powers even without the presence of judicial councils. Third, we have also observed a trend towards increasing regulation in the field and delegation of newly regulated governance competences mostly to judges—again, this trend did not correlate with the existence of judicial councils. In other words, increasing regulation of judicial governance contributes to judicial empowerment.

The JSG Index informs us about the relationship between the institutional organization of judicial governance and the empowerment of judges. The preliminary results of the JSG Index have significant scholarly and policy implications. JSG can exist outside the judicial council model. While more theorizing on the relationship between empowerment and judicial insulation will be needed, the preliminary findings are important for transitional or highly polarized societies, where judicial councils are proving to be problematic and prone to attracting politicization. The JSG Index also allows us to measure more precisely how much power judges actually have in individual types of judicial councils—making the scholarship on the institutional position of judicial councils more precise.

Apart from the comprehensive and vigorous testing of the JSG Index in numerous jurisdictions, the next step in future research should be a careful empirical analysis of the relationship between the allocation of governance powers to judges and the outcomes of judicial governance (particularly with regard to judicial independence, accountability, public confidence in the courts, and courts' efficiency). Another future line of research could discuss the resistance of various institutional arrangements to judicial corruption, different channels of politicization in systems with low and high JSG, or the degree of political capture of the judiciary in relation to increased regulation and judicial empowerment. Instead of dwelling on institutional models, which are often simplified and do not capture the full complexity of judicial governance, the JSG Index will allow us to understand whether there is a relationship between the balance of power between judges and politicians and the performance of the courts, broadly understood.

## Supporting information


**Appendix S1:** Supporting information

## Data Availability

The data that support the findings of this study are available from the corresponding author upon reasonable request.

## Endnotes

^1^
See, for example, Consultative Council of European Judges, Opinion no. 10 (2007) on the Council for the Judiciary at the service of society (https://rm.coe.int/168074779b).

^2^
See Consultative Council of European Judges, Opinion no. 24 (2021) on the evolution of the Councils for the Judiciary and their role for independent and impartial judicial systems (https://rm.coe.int/opinion-no-24-2021-of-the-ccje/1680a47604); judgment of the European Court of Human Rights, *Anželika Šimaitienė v Lithuania*, No. 36093/13; and judgment of the Court of Justice of the European Union, *A.K*., C‐585/18. Note that both the ECtHR and the CJEU held that the involvement of a body like a judicial council or a judicial appointments committee is in principle likely to make the appointments process more objective (AK (C‐585/18) EU:C:2019:982 at [137]; Anželika Šimaitienė v Lithuania (36093/13) 21 April 2020 at [82].

^3^
World of Rule of Law Index, Latent JI, Polity IV. World Economic Forum.

^4^
For one of the few exceptions see the definition of judicial governance offered by Castillo‐Ortiz as a “set of institutions, rules, and practices in a jurisdiction that organize, facilitate, and regulate the exercise by the judicial branch of its function of the application of law to concrete cases.” Nevertheless, Castillo‐Ortiz (2019) relies on the element of self‐governance, that is, the exercise of judicial governance by the judicial power.

^5^
The questionnaire can be accessed at https://pgwrk-websitemedia.s3.eu-west-1.amazonaws.com/production/pwk-web-encj2017-p/Projects/Indicators_Questionnaire_Scoring.pdf.

^6^
See the European Network of Councils for the Judiciary—Performance Indicators 2017 (https://www.encj.eu/images/stories/pdf/workinggroups/independence/encj_report_ia_ga_adopted_ga_13_6.pdf). Compare also Van Dijk & Vos, 2018.

^7^
See parts 3.3.2 of different editions of the EU Justice Scoreboard (https://ec.europa.eu/info/policies/justice-and-fundamental-rights/upholding-rule-law/eu-justice-scoreboard_en).

^8^
See paragraph 1 of the 2008 Resolution on Self Governance for the Judiciary (https://www.encj.eu/images/stories/pdf/opinions/budapestresolution.pdf), or the 2017 Final Report of the ENCJ's Project Group on the Distillation of ENCJ Guidelines Recommendations and Standards (https://www.encj.eu/images/stories/pdf/workinggroups/encj_distillation_report_2004_2017.pdf).

^9^
See arts. 9 and 13 of the 2010 Magna Carta of Judges (https://rm.coe.int/2010-ccje-magna-carta-anglais/168063e431).

^10^
See art. 32 of the 2010 Report on the Independence of the Judicial System (https://www.venice.coe.int/webforms/documents/default.aspx?pdffile=CDL-AD(2010)004-e).

^11^
See art. 41 of the 2010 Recommendation CM/Rec(2010)12 of the Committee of Ministers to member states on judges **(** https://search.coe.int/cm/Pages/result_details.aspx?ObjectID=09000016805afb78 **).**

^12^
See arts. 2.3 and 3 of the 1999 Universal Charter of the Judge (https://www.iaj-uim.org/universal-charter-of-the-judge-2017/).

^13^
For a more detailed explanation of the sources of data analysis, as well as the rationales behind the choice of de jure index, see Appendix, Section 2. B.

^14^
The obligatory consultation means that the law conditions the adoption of new amendments with an official consultation of judges. The competence secures that judges are heard and can comment on important legislation related to courts and that the nonjudges adopting the decision cannot circumvent them.

^15^
In order fully to understand the autonomy of judges in the field of judicial governance it is also important to analyze who these non‐judges are, with particular focus being placed on the role of autonomous expert and judicial governance bodies which might be characterized as fourth‐branch institutions (for more on this topic see Khaitan, 2022, Tushnet, 2021). Applying the fourth‐branch scholarship in then judicial governance field, we provide an in‐depth analysis of these autonomous actors in Authors: Judicial Councils as Forth Branch Institutions. 2022 forthcoming.

^16^
One of the exeptions was introduced in 2020 in Slovakia, where the constitutional amendment (423/2020 Col.) provided that the Parliament can remove a member of a judicial council by a simple majority.

^17^
For the consistency of our approach with existing scholarship see also Elsig and Pollack (2014).

^18^
These are persons responsible for the organizational, personnel, financial, administrative and other technical running of the particular court. Typically, these position are found at apex courts.

^19^
The data were coded by authors and cross‐validated in the research team. Hand‐coded datasets were double‐checked by country experts.

^20^
For every year we coded the state of legislation as of 1 January.

^21^
The Constitution does not specify an exact number; the composition of the CSM has changed in the past. Since 2002 it has settled at 27.

^22^
See also indicators in World Rule of Law Index 2020, V‐Dem, or Polity IV dataset.

^23^
The existing hypotheses do not, however, relate increase institution building with better performance.

^24^
On the contrary, regulatory dimension seems to be of particular importance in the case of international courts, which typically shape their own rules of procedure (Tsereteli & Smekal, 2018, p. 2144).
