# Supplementary material for: Judicial Self‐Governance Index: Towards better understanding of the role of judges in governing the judiciary
Source: Regul Gov. 2022 Jan 4;17(1):22–42. doi: 10.1111/rego.12453 (PMC10078789; doi:10.1111/rego.12453)
Supplement: Supplementary file 1 — Appendix S1: Supporting information [file REGO-17-22-s001.docx]

Appendix

Judicial Self-Governance Index

# 1. Introduction

The JSG index measures the amount of power held by judges in competences related to judicial governance (i.e. the extent of judicial self-governance).

It builds on a broad definition of judicial self-governance, which we define as a structured model of social coordination which produces and implements a set of institutions, rules and practices which are collectively binding and which regulate how the judicial branch exercises its functions (Börzel & Risse 2010, Castillo-Ortiz 2019, Kosař 2018). Judicial self-governance (JSG) then captures the extent to which judges and courts participate in judicial governance.

The chosen definition thus allows us to measure the allocation of judicial governance powers to judges in any institutional regulatory system. It is applicable to countries with judicial councils (and various types of judicial councils), court presidents, the court service, or specialized domestic judicial appointment commissions. We also believe that the JSG Index could easily be amended in future to cover also international judicial governance models such as the Article 255 TFEU Panel for the selection of Court of Justice judges and the Committee of Ministers’ (CM) Advisory Panel of Experts on Candidates for Election as Judge to the ECtHR.

# 2. Coding and Data Aggregation

## A. Unit of analysis

The unit of our analysis is legal competence related to judicial governance. Drawing on Kosař‘s previous work (2018), we divided judicial governance into 8 dimensions: regulatory, administrative, personal, financial, educational, informational, digital and ethical. Next, we identified relevant competences for each of the dimensions based on existing literature (Cukierman 1992; Feld and Voigt 2003; Hanretty 2009; Hayo & Voigt 2014; Voigt et al. 2015; Hayo & Voigt 2016; Juwayeyi 2017; Gutmann & Voigt 2018; Van Dijk & Vos 2018), data collected by CEPEJ and EU Justice Score, other indexes measuring judicial independence, accountability, effectiveness of courts and related values, as well as indexes targeting other governance actors (central banks, regulatory agencies, etc.). Lastly, the set of competences was also influenced by expert knowledge and country-specific reports addressing judicial governance in individual European jurisdictions (German Law Journal, special issue 19, 2018). In what follows we offer a comprehensive list of dimensions and respective competences, which are then explained in more detail in section 3.

**I. Regulatory competences**

1. establishment of a court
2. abolition of a court (also by merger)
3. jurisdiction of a court
4. court statute
5. legal procedural rules

**II. Administrative competences**

1. composition of a court - overall number of judges
2. composition of a court - structure (number of panels)
3. composition of a court - personnel (composition of panels)
4. legal questions/issues resolved by individual panels within a court
5. number of administrative personnel
6. number of law clerks
7. initial case assignment
8. case reassignment (in the event of illness, removal from office etc.)
9. evaluation of a court (decision-making, financing, budget, etc.)

**III. Personal competences**

1. selection of a judge (selecting one applicant for an open seat)
2. appointment of a judge
3. temporary removal of a judge
4. removal of a judge
5. compulsory retirement age
6. transfer of a judge to another court with her consent (relocation)
7. temporary transfer of a judge to another court with her consent (secondment)
8. transfer of a judge to a higher court with her consent (promotion)
9. transfer of a judge to a lower/other court without her consent (demotion or transfer without consent)
10. temporary transfer of a judge out of the judiciary
11. initiation of disciplinary action against a judge
12. decision in disciplinary proceedings
13. authorization of the criminal prosecution of a judge
14. authorization of proceedings on the civil liability for judicial activities
15. reviewing complaints against a judge
16. evaluation of a judge
17. financial sanction against a judge (temporary salary reduction, fine, pension reduction, etc.)
18. financial bonus for a judge (discretionary)
19. reprimand of a judge

**IV. Financial competences**

1. size of a court's budget
2. a court's budget allocation
3. salary of a judge - fixed component (across the board)
4. salary of a judge - discretionary component (across the board)

**V. Educational competences**

1. compulsory education/training of judicial candidates – structure
2. compulsory education/training of judicial candidates – content
3. certification of a judge (giving them the opportunity possibility to apply for an open position)
4. further education/training of judges – structure
5. further education/training of judges – content

**VI. Informational competences**

1. determining which judgements are published
2. allowing the recording of trials
3. existence of obligation to disclose judges' property
4. existence of obligation to publish the list of judges (and their CVs, clerks)
5. existence of obligation to disclose judges’ political affiliations
6. existence of obligation to publish continuous statistics on caseload (coming, pending, decided)
7. existence of obligation to publish the annual report on a court
8. existence of obligation to publish the annual report on the whole of the judiciary
9. existence of obligation to publish the case assignment system (panels composition, work schedule)

**VII. Digital competences**

1. (physical) placement of servers (i.e. place where data - e.g. digital case files - are stored)

**VIII. Ethical competences**

1. preparation of the code of conduct
2. interpretation of the code of conduct (e.g. advising judges on how to behave via "hotlines")
3. authorization of judges to communicate with media
4. explaining decisions
5. regulation of off-bench activities - teaching
6. regulation of off-bench activities - party membership
7. regulation of off-bench activities - political engagement
8. regulation of the business activities of judges

## B. Sources

The JSG Index measures only *de jure* competences, i.e. it looks at the distribution of competences as stipulated by the law. “The law” in our understanding includes five types of sources: constitutions, organic laws (i.e. laws forming the basis of a state’s government and governance), ordinary legal statutes, sub-statutory legal acts (i.e. subordinate legislation issued by an entity under a power delegated by the Parliament; typically, this would be more technical or detailed legislation issued by the ministry of justice which, however, is still generally binding) and the case law (i.e. interpretation of and filling the gaps between the written legal norms). In order to collect the data, we therefore examine these five sources in the country of interest in the year in question (i.e. including various amendments, interpretations, etc. valid in that year).

In other words, the JSG index does not capture the *de facto* execution of judicial governance competences. Reflecting on the existing scholarship on the relationship between *de jure* and *de facto* indicators, particularly those related to judicial independence (Hanretty 2009; Juwayeyi 2017; Gutmann & Voigt 2018, Hayo & Voigt 2021), the *de facto* dimension of the index is planned to be added in future. As of now however, the JSG index does not contain it and focuses on the *de jure* level. We believe that an understanding of the structural distribution of competences on the institutional, formal level is a self-standing element in broader research on the empowerment of judges under different models of judicial governance. Furthermore, the formal *de jure* analysis is gaining in importance, with the most recent case law of both European supranational courts (European Court of Human Rights and Court of Justice of the European Union) addressing formal requirements of judicial independence. In the face of constitutional crises in Poland, Hungary and Romania, both Courts have demonstrated increasing focus on and requirements for the establishment of judicial councils, equipped with a certain set of competences vested in the hands of judges (see e.g. European Court of Human Rights, *Thiam v France*, 80018/12, Judgment of 18 October 2018, and Court of Justice of the European Union, C‑83/19, C‑127/19, C‑195/19, C‑291/19 and C‑355/19 and C‑397/19, AFJR and others, judgement of 18 March 2021). The JSG Index allows us to measure this formal delegation of power upon judges and allows us to explore the broader question whether judicial councils deliver in this respect and what other models might allow self-governance.

That said, we aspire to add the measurement of *de facto* execution of individual judicial governance competences to the JSG Index in future.

## C. Actors

The JSG Index measures competences given to judges (or, in other words, the share of judicial governance powers *formally held* by judges). In doing so the JSG Index implements a logical reduction of the plethora of actors involved in JSG into two groups: judges and non-judges (politicians, lawyers, experts). While this dichotomy is helpful in the analysis and juxtaposition of power directly held by judges against that of other actors (i.e. what share of all existing judicial governance is held by judges), it becomes slightly complicated when we take into account the participation of multi-actor JSG bodies, such as judicial councils or court services, which are composed of both judges and non-judges.

Before we can explain the process how we determined the degree of involvement of judges and non-judges in judicial governance, we must briefly address who is a “judge” and who is a “non-judge”. While the distinction between judges and non-judges is obvious in most cases, there are some borderline examples such as part-time judges, lay judges), or prosecutors (who are both *de jure* and *de facto* understood as members of the judiciary in some jurisdictions like Italy). Regarding the two first examples, our answer is simple and straightforward. For the purpose of our JSG Index, a “judge” means a full-time professional judge. A separate conceptual issue, which we refer to as “judicial nesting”, arises, when the jurisdiction allows for temporary assignment of judges to the Ministry of Justice and/or other governmental and parliamentary bodies. In other words, we have a full-time professional judge, whose office is suspended, and she serves temporarily at the other branch of government. We use the same rule of dependency to code such cases. This means that while the identity of actors who *de facto* influences the decisions taken on behalf of the Ministry of Justice is important for understanding the actual state of judicial governance, it is the leadership of the Ministry of Justice (i.e. non-judges) who *de jure* issue instructions on how the Ministry acts substantively, even if politicians at the helm of the Ministry listen to the advice provided by judges temporarily assigned to the Ministry. In other words, our JSG Index captures only the *de jure* powers vested in the hands of judges across different models of judicial governance and a more nuanced analysis of the *de facto* division of power in various dimensions of judicial governance is the task for in-depth case studies. A “non-judge” is then defined as anyone who is not a “judge”. As we are aware that there is a variety of “non-judges” involved in judicial governance (politicians, members of other legal professions, civil society, and experts such as human resource specialists or economists) and that each group of these “non-judges” can raise specific problems, the JSG Index contains also data on who these non-judges are, which might serve for future analyses of how various actors constrain judicial autonomy and independence.

In order truthfully to capture the influence of judges on the implementation of each competence, we therefore carried out a three-step process. First, we indicated all the actors participating in each judicial governance competence. These typically include presidents, ministers of justice, court presidents and court vice-presidents, directors of courts, judicial councils, judicial associations, judicial academies. Secondly, we analysed the composition of every actor (in every specific country in the relevant year). This was fairly easy for individual actors such as the court president, minister of justice or the presidents. However, things got more complicated with the introduction of multi-actor JSG bodies, judicial councils in particular. For every multi-actor JSG body we asked whether judges inside that body have a decisive majority or have to decide together with their non-judge peers. This was evaluated on the basis of both the composition of the body and the rules of its decision-making. If the multi-actor JSG body consisted mostly of judges and these judges had enough voting power to implement a decision (form a purely judicial coalition), it was considered as fully controlled by judges and assigned a value of 1. Similarly, if the multi-actor JSG body was composed mostly of non-judges it was assigned a value of 0. The value of 0.5 was assigned to balanced multi-actor JSG bodies where neither group could have implemented a majoritarian decision without the support of members of the opposite camp.

For example, if the judicial council consisted of 11 members, 6 judges and 5 non-judges, and adopted decisions with a 2/3 majority, we would evaluate the decision-making power of the judges as 0.5 (as judges need to negotiate with their non-judicial peers).

The third and final step consisted of assigning the score to each individual competence, averaging the involvement of judges and non-judges across all the JSG actors participating in the competence we wanted to measure. To do so, we took into consideration both the scores assigned to each JSG actor and the rules on decision-making for the given competence.^[[1]](#footnote-1)^

It is, however, important to note that our conceptualization of JSG competences does not cover the question of how individual JSG bodies emerge. We are merely listing competences related to the actual execution of competences given to judges. This differentiation is highlighted especially in a personal dimension of JSG. It is quite common to regulate the appointment and removal of court presidents separately from those of rank and file judges. While it might be tempting to include both processes in a list of JSG competences, they address a different question. Court presidents are one of the JSG bodies. The inclusion of specific procedures related to their managerial, and not their judicial, role would mean that we are mixing competences related to judicial governance with competences and rules related to the existence and functioning of JSG bodies. We acknowledge that the composition, role and legitimacy of individual JSG bodies, judicial councils and court presidents in particular plays an important role in the overall picture of JSG, and might influence the vigour with which these bodies execute their competences *de facto*. However, we consider this a separate question and do not include it in the *de jure* JSG index. ^[[2]](#footnote-2)^

## D. Scale for JSG Index scoring

The assessment of the extent of (decision-making) power held by judges in every judicial governance competence is measured on the following scale (Table A.1):

| \| **Table A.1** \| \| \| --- \| --- \| \| **To what extent are judges de jure involved in deciding on a competence?** \| **Value** \| \| judges decide \| 1 \| \| judges or non-judges decide (independently of each other) \| 0.75 \| \| judges negotiate with non-judges (both have veto power) \| 0.5 \| \| judges are consulted \| 0.25 \| \| not at all \| 0 \| |  |
| --- | --- | --- | --- | --- | --- | --- | --- | --- | --- | --- | --- | --- | --- | --- | --- |

If the law stipulates that a certain competence belongs to judges exclusively (typically a competence held by a court, court president, disciplinary panel composed of judges only), we code it 1.

If the law stipulates that either judges or non-judges decide **independently of each other (one or both can decide)**, we code it 0.75. This covers situations such as the initiation of disciplinary proceedings, which can be done either by the Minister of Justice or by a court president.

If judges negotiate with non-judges and both have a veto power, we code it 0.5. This scenario includes a situation where a judge needs the approval of the Minister of Justice to be transferred and, at the same time, the Minister of Justice cannot transfer her against her will.

If the law presumes that judges have to be consulted on the execution of a certain competence, we code this 0.25. For example, courts (i.e. judges) often take part in the legislative process regarding competences of the judiciary, procedural codes, etc. They do not have a veto power, nor do they decide, but they have to (or can) be consulted, which gives them a channel through which to express their views and opinions.

If the competence is held exclusively by non-judges, we code this 0. For example, in Czechia judges are appointed by the President.

## E. Alternatives on aggregating results in JSG index.

Our conceptualization of judicial self-governance consists of eight dimensions. In these eight dimensions, we introduced altogether sixty indicators aimed at measuring the extent of judicial self-governance. Approaching the JSG Index as a compensatory concept has its benefits (it allows us to capture transparently what is happening inside individual judicial governance dimensions) and drawbacks (less salient competences, which might also be easier to delegate to judges, make it seem there is a lot of judicial self-governance). Nevertheless, judicial governance is a growing field. New dimensions which were not sufficiently explored by the empirical or theoretical scholarship are appearing and any pre-emptive weights assigned to them in order to create a non-compensatory model would be even more normative than in the compensatory model.

Moreover, the way we have organised individual dimensions of judicial self-governance and identified competences *within* those dimensions incidentally increases the importance of the personal dimension (compared to the other dimensions of judicial self-governance), as that dimension contains the most competences (19), and is also typically identified by the scholarship as a crucial one for judicial governance. Simple aggregation on the level of dimension and the cross-dimensional comparison of JSG competences therefore allows us to achieve results comparable with recent theoretical scholarship; to observe whether the personal dimension behaves differently compared to other parts of judicial governance; and to correlate each dimension individually with indicators of independence, accountability or efficiency.

There were, however, several other alternatives for aggregating the data. After the data collection was completed, we faced two major crossroads that forced us to make methodological choices about how to aggregate the index: (A) whether or not to include dimensions; and (B) whether or not to include default values for indicators.

This left us with at least five alternatives for how to aggregate the value of the JSG index for any given country at any given point in time. In addition, the inclusion of dimensions in the aggregation process and refraining from using default values allows us to calculate a JSG index score with the use of dimensions’ relative weights (Table A.2). Based on the consideration analysed below, we decided to implement alternatives A and B in the JSG Index presented in main article.

| **Table A.2** | | | | |
| --- | --- | --- | --- | --- |
|  |  | **Dimensions** | | |
|  |  | **Not included** | **Included** | |
|  |  |  | Absolute weight | Relative weight |
| **Default values** | **Not included** | **A** | **C** | **E** |
|  | **Included** | **B** | **D** | |

1. A JSG index without dimensions and without default values

$$JSG= \frac{q_{1}+q_{2}+\left( \ldots\right)+q_{60}}{n},$$

where $q_{x}$ refers to individual indicators and where each $q_{x}$ = [0, 1] or is coded as *NA* or a missing value. As stated before, there are sixty indicators in the JSG index; therefore *n* equals the number of indicators that were coded without the missing values.

1. A JSG index without dimensions and with default values

$$JSG= \frac{q_{1}+q_{2}+\left( \ldots\right)+q_{60}}{N},$$

where $q_{x}$ refers to individual indicators and where each $q_{x}$ = [0, 1]. For values that in model A were coded as *NA*s, in this mode of aggregation missing values are replaced by a value of 0 or 1. For more see Appendix 1. As there are sixty indicators, $N=60$.

1. A JSG index with dimensions and without default values

$$JSG= \frac{D_{1}+D_{2}+\left( \ldots\right)+D_{8}}{y},$$

where $D_{x}$ refers to the score on individual dimensions, where each $D_{x}=\left[ 0, 1 \right]$ is based on the following calculation:

$$D_{x}=\frac{q_{i}+\left( \ldots\right)+q_{n}}{n_{D_{x}}},$$

where $q_{i}+\left( \ldots\right)+q_{n}$refers to the sum of the values of all indicators in a dimension $D_{x}$, and where $n_{D_{x}}$ refers to a total number of indicators in a dimension $D_{x}$ without those that were coded as *NA*s or missing values.

Finally, $y$ refers to a number of dimensions where at least one indicator was coded on a scale from 0 to 1, so that not all indicators in the particular dimension were coded as *NA*s.

1. A JSG index with dimensions and with default values

$$JSG= \frac{D_{1}+D_{2}+\left( \ldots\right)+D_{8}}{Y},$$

where $D_{x}$ refers to the score for individual dimensions, where each $D_{x}=\left[ 0, 1 \right]$ is based on the following calculation:

$$D_{x}=\frac{q_{i}+\left( \ldots\right)+q_{n}}{N_{D_{x}}},$$

where $q_{i}+\left( \ldots\right)+q_{n}$refers to the sum of the values of all indicators, including those coded with default values, in a dimension $D_{x}$, and where $N_{D_{x}}$ refers to a total number of indicators in a dimension $D_{x}$.

Unlike in Model C, here $Y$ refers to a total number of dimensions, hence $Y=8$.

1. A JSG index without default values using relative weights of dimensions

$$JSG=\frac{\left( D_{1}*W_{1} \right)+\left( \ldots\right)+ \left( D_{8}*W_{8} \right)}{n},$$

where $D_{x}$ refers to the score for individual dimensions, where each $D_{x}=\left[ 0, 1 \right]$ is based on the following calculation:

$$D_{x}=\frac{q_{i}+\left( \ldots\right)+q_{n}}{n_{D_{x}}},$$

where $q_{i}+\left( \ldots\right)+q_{n}$refers to the sum of the values of all indicators in a dimension $D_{x}$, and where $n_{D_{x}}$ refers to the total number of indicators in a dimension $D_{x}$ without those that were coded as *NA*s or missing values.

$W_{x}$ refers to the weight of a dimension $D_{x}$ which is expressed as a total number of indicators on each dimension. $n$ refers to the sum of the total number of indicators in dimensions with at least one indicator coded on a scale from 0 to 1, hence without NAs or missing values.

Comparisons of the JSG Index scores after the implementation of each of the alternatives are included in Table A.2.

| **Table A.3** | | | | | | |
| --- | --- | --- | --- | --- | --- | --- |
| **Country** | **Year** | **Model A** | **Model B** | **Model C** | **Model D** | **Model E** |
| **Czechia** | **1994** | 0.196 | 0.563 | 0.157 | 0.591 | 0.391 |
|  | **1999** | 0.188 | 0.504 | 0.126 | 0.538 | 0.328 |
|  | **2004** | 0.358 | 0.608 | 0.286 | 0.522 | 0.47 |
|  | **2009** | 0.308 | 0.558 | 0.259 | 0.495 | 0.416 |
|  | **2014** | 0.304 | 0.554 | 0.251 | 0.487 | 0.419 |
|  | **2019** | 0.325 | 0.575 | 0.273 | 0.509 | 0.433 |
| **Germany** | **1994** | 0.288 | 0.588 | 0.198 | 0.558 | 0.364 |
|  | **1999** | 0.288 | 0.588 | 0.198 | 0.558 | 0.364 |
|  | **2004** | 0.288 | 0.588 | 0.198 | 0.558 | 0.364 |
|  | **2009** | 0.288 | 0.588 | 0.198 | 0.558 | 0.364 |
|  | **2014** | 0.288 | 0.588 | 0.198 | 0.558 | 0.364 |
|  | **2019** | 0.321 | 0.604 | 0.213 | 0.571 | 0.517 |
| **Italy** | **1994** | 0.5 | 0.75 | 0.417 | 0.662 | 0.72 |
|  | **1999** | 0.5 | 0.75 | 0.417 | 0.662 | 0.72 |
|  | **2004** | 0.5 | 0.75 | 0.417 | 0.662 | 0.72 |
|  | **2009** | 0.567 | 0.817 | 0.56 | 0.762 | 0.805 |
|  | **2014** | 0.567 | 0.817 | 0.56 | 0.762 | 0.805 |
|  | **2019** | 0.567 | 0.817 | 0.56 | 0.762 | 0.805 |
| **Slovakia** | **1994** | 0.125 | 0.525 | 0.081 | 0.539 | 0.292 |
|  | **1999** | 0.133 | 0.517 | 0.091 | 0.542 | 0.295 |
|  | **2004** | 0.354 | 0.588 | 0.315 | 0.617 | 0.489 |
|  | **2009** | 0.408 | 0.575 | 0.332 | 0.489 | 0.49 |
|  | **2014** | 0.433 | 0.6 | 0.34 | 0.497 | 0.509 |
|  | **2019** | 0.454 | 0.588 | 0.348 | 0.477 | 0.502 |

# 3. Coding instructions

**I. Regulatory competences**

*These competences relate to the basic existence of the judiciary, typically regulated in the Constitution and specific Statutes on Courts and Judges. Naturally, the regulatory competences have to be wielded mainly by non-judges (i.e. Parliament as the core legislator), as they include core constitutional decisions (establishment of courts, outlining of their jurisdiction, etc.). However, the regulation of courts does change over time, and we are interested to see whether judges are participating in the drafting of potential future legislative amendments of regulatory competences, at least in a consultative capacity.*

1. Establishment of a court

*This competence covers decisions on the creation of new courts.*

1. Abolition of a court (also by merger)

*This competence covers the abolition of existing courts, even if this may result from the merger of two or more courts, or abolishing and establishing a new court.*

1. Jurisdiction of a court

*This competence covers the regulation of a court’s jurisdiction: territorial, material (scope of legal questions and issues) and functional (first, second, third instance, appellate courts, etc.) competences.*

1. Court statute

*This covers the question of who has the competence to establish the court’s statute. Although we presume that this competence will in the majority of cases belong to the legislator, we also expect that judges may be consulted on the creation/amendment of the statute in some jurisdictions. The competence to issue a court statute is particularly relevant for supranational judicial bodies.*

1. Legal procedural rules

*This is a competence to set internal procedural rules for courts' decision-making. As with the last competence, we expect judges to have at least consultative powers in some jurisdictions.*

**II. Administrative competences**

*This set of competences covers the functioning of courts, their composition, their case assignment system and the evaluation of their activity.*

1. Composition of a court - overall number of judges

*We are interested in the extent to which, according to the law, judges decide how many judges sit at the court.*

1. Composition of a court - structure (number of panels)

*We are interested in the extent to which, according to the law, judges decide on the internal structure of the court, particularly the number of its panels. In the case of apex courts, we are* ***not interested in divisions****, only in panels.*

1. Composition of a court - personnel (composition of panels)

*We are interested in the extent to which, according to the law, judges decide on the composition of panels, i.e. how many and which judges sit on the panel. The number of judges on the panel influences the rotation of certain legal issues: in other words, how many judges decide certain topical legal questions.*

1. Legal questions/issues solved by individual panels within a court

*We are interested in the extent to which, according to the law, judges decide on the number of panels that can decide the same legal questions/issues, i.e., whether, for example, state liability is in the discretion of 1, 2, 3…panels. In other words, this competence influences how many and which judges can potentially decide the case.*

1. Number of administrative personnel

*We are interested in the extent to which, according to the law, judges decide on the number of administrative personnel hired by the court.*

1. Number of law clerks

*We are interested in the extent to which, according to the law, judges decide on the number of law clerks employed by the court.*

1. Initial case assignment

*Initial case assignment can be the prerogative of the president of the court, can be done via an algorithm set by the Ministry or, typically, be done randomly by the court’s registry, while respecting the working schedule of the court and its judges (this goes for the algorithm as well). If it is set by the registry, we code it 0.*

1. Case reassignment (in the event of illness, removal from office etc.)

*In this category we want to know if the president or a different judge has the competence to take the case and assign it to someone else.*

1. Evaluation of a court (decision-making, finances, budget, etc.)

*The coding includes the competence of both continuous and random evaluation of the court as a whole (speed and quality of decision-making, finances, budget, etc.).*

**III. Personal competences**

*This set of competences covers decisions on the selection, appointment, removal, career and disciplining of judges.*

1. Selection of a judge (selecting one applicant for a vacancy)

*We are interested in the extent to which, according to the law, judges decide on the selection processes for applicants for a judicial vacancy.*

1. Appointment of a judge

*We are interested in the extent to which, according to the law, judges take part in the appointment of a judge. While the selection presumes competition for a judicial vacancy, appointment is a formal constitutive process.*

1. Temporary removal of a judge

*We are interested in the extent to which, according to the law, judges can temporarily remove a judge from her position.*

1. Removal of a judge

*We are interested in the extent to which, according to the law, judges can permanently remove a judge from her position. Typically, competences 17 and 18 will both be the result of disciplinary proceedings. In such a case, we take into account the composition of disciplinary panels.*

1. Compulsory retirement age

*This competence covers the extent to which judges may decide on what the compulsory retirement age will be.*

1. Transfer of a judge to another court with her consent (relocation)

*We are interested in the extent to which, according to the law, judges may decide to transfer a judge, at her request, to a different court. The judge’s consent is needed.*

1. Temporary transfer of a judge to another court with her consent (secondment)

*We are interested in the extent to which, according to the law, judges may decide* ***temporarily*** *to transfer a judge from her position to a different court. This reassignment might include an internship at an international judicial institution or foreign court. The judge’s consent is needed.*

1. Transfer of a judge to a higher court with her consent (promotion)

*We are interested in the extent to which, according to the law, judges may decide to transfer a judge to a higher-ranking court, e.g. from a court of first instance to the appellate court, but not the apex court. In the event that the same competence relates to apex courts, we are interested in the extent to which, according to the law, judges may decide to transfer a judge to the apex court. The judge’s consent is needed.*

1. Transfer of a judge to a lower/different court without her consent (demotion or transfer without consent)

*We are interested in the extent to which, according to the law, judges may decide to transfer a judge against her will to a court of the same or a lower instance, e.g. from the appellate court to a court of first instance.* ***The consent of the transferred/demoted judge is not needed.***

1. Temporary transfer of a judge out of the judiciary

*We are interested in the extent to which, according to the law, judges may decide temporarily to transfer a judge out of the judiciary – this typically includes internships at ministries of justice, etc.*

1. Initiation of disciplinary action against a judge

*We seek to know to what extent judges initiate, i.e. propose, disciplinary proceedings against judges. In many jurisdictions, this competence is shared between many actors. In Czechia, for example, it is for court presidents (i.e. judges), the minister of justice, the President of the republic and the Public Defender of Rights to initiate the proceedings. Since each of them can initiate the action independently, we code this as 0.75.*

1. Decision in disciplinary proceedings

*We seek to know to what extent judges take part in decision-making in disciplinary proceedings. Typically, this question will be aimed at the composition of disciplinary panels.*

1. Authorization of the criminal prosecution of a judge

*We seek to know who has the competence to authorize the criminal prosecution of a judge.*

1. Authorization of proceedings regarding civil liability for judicial activities

*In this question we are interested in the extent to which judges can decide that a judge will be held liable for her decision-making. In some jurisdictions, the proceedings might be initiated by the Ministry of Justice. If the disciplinary panel finds a judge liable, the Ministry may consequently demand compensation from that judge. Given the complexity of the interaction between actors, we code such a situation as 0.5.*

1. Reviewing complaints against a judge

*To what extent do judges decide on complaints submitted against judges by individuals? Is it a court president who decides? Or a Minister of Justice? Note: in a scenario where a court employee (non-judge) appointed by the court president decides on complaints in the first instance, we code this as 1.*

1. Evaluation of a judge

*To what extent do judges take part in the evaluation of individual judges? (In other words, if a judge is being evaluated, who, according to the law, decides and makes the evaluation?)*

1. Financial sanction against a judge (temporary salary reduction, fine, pension reduction, etc.)

*To what extent, according to the law, can judges impose financial sanctions on a judge? By financial sanction we mean a salary reduction (usually of a temporary character), a fine, reduction of a pension, etc.*

1. Financial bonus for a judge (discretionary)

*To what extent, according to the law, can judges decide to award a judge a financial bonus?*

1. Reprimand of a judge

*Compared to point 31, this competence covers non-monetary sanctions against a judge.*

**IV. Financial competences**

*This set of competences covers the financial administration of courts, i.e. decisions on their budgets, judicial salaries, etc.*

1. Size of a court's budget

*To what extent, according to the law, do judges decide on the size of the (apex) court’s budget? We are also interested in their part in possible negotiations.*

1. A court's budget allocation

*Compared to competence 34, the budget allocation assesses the allocation of money to individual parts of the budget. Typically, the budget of a court will be divided into personal costs (salaries), material costs (furniture, cars, computers, etc.), services (online servers, websites, proof reading, translation services, etc.) or, even more specifically, the courts may assign money specifically to one issue (roof repairs, etc.). We are interested in the extent to which judges can decide on moving money freely between these categories.*

1. Salary of a judge - fixed component (across the board)

*This is the core part of a judge’s salary.*

1. Salary of a judge - discretionary component (across the board)

*This is part of a salary, not a random bonus. The discretionary part of the salary cannot later be removed; it is paid monthly once it is awarded to a judge.*

**V. Educational competences**

*This set of competences covers the education and training of judges.*

1. Compulsory education/training of judicial candidates – structure

*Compulsory education covers the legal education necessary for one to qualify for a future position as judge. It includes both university education and its structure (e.g. type of university degree) and the further education of judicial candidates before their appointment, if applicable.*

1. Compulsory education/training of judicial candidates – content

*This competence covers the extent to which judges, according to law, decide* ***on the content*** *of the legal education necessary for the future position of a judge.*

1. Certification of a judge (giving them the opportunity to apply for a vacancy)

*Certification of a judge relates to the setting of requirements one must fulfil in order to be eligible to apply for a judicial vacancy. Certification is typically a judicial exam; in many jurisdictions the judicial exam might be exchanged for another profession exam (attorney, etc.). If judges sit on at least one existing exam committee which organises the exam, we code the competence as 1 (i.e. this also covers a situation where the judge can obtain certification via the attorney’s exam organised by a committee of attorneys, given that the attorney’s exam is an alternative to an existing judicial exam organised by a committee composed of judges).*

1. Further education/training of judges – structure

*This competence covers education and training after judicial appointment, i.e. that of already appointed judges. We are interested in the* ***structure of the education.***

1. Further education/training of judges – content

*This competence covers education and training after judicial appointment, i.e. that of already appointed judges. Here, we are interested in the* ***content of further education.***

**VI. Information**

*This set of competences covers the publication and accessibility of information on the courts, their activity, composition and case law.*

1. Determining which judgements are to be published

*We are interested in who formally regulates if and which judgements and decisions should be published (usually in online databases). We are also interested in who should publish (i.e. whether judges) these decisions.*

1. Allowing the recording of trials

*We are interested in whether judges, according to the law, take part in decisions allowing the recording of trials.*

1. Existence of an obligation to disclose judges' property

*If there is such an obligation, does the law assign it to judges (is it a judge who must require the disclosure of judges’ property?)?*

1. Existence of obligation to publish the list of judges (and their CVs, clerks)

*If there is such an obligation, does the law assign it to judges (is it a judge who must publish it?)?*

1. Existence of an obligation to disclose judges’ political affiliation

*If there is such an obligation, does the law assign it to judges (is it a judge who must require the disclosure?)?*

1. Existence of an obligation to publish continuous statistics on caseload (coming, pending, decided)

*If there is such an obligation, does the law assign it to judges (is it a judge who publishes it? We expect this competence to be typically held by ministries of justice, i.e. 0 in coding).*

1. Existence of an obligation to publish the annual report on a court

*If there is such an obligation, does the law assign it to judges (is it a judge who publishes it?)?*

1. Existence of an obligation to publish the annual report on the national judiciary

*If there is such an obligation, does the law assign it to judges (is it a judge who publishes it?)? Compared to competence 49, this will include a report on all courts, ordinary and apex.*

1. Existence of an obligation to publish the case assignment system (panels’ composition, work schedule)

*If there is such an obligation, does the law assign it to judges (is it a judge who publishes it?)?*

**VII. Digital competences**

*This competence covers digital aspects of courts’ functioning.*

1. (Physical) placement of servers (i.e. the place where data - e.g. digital case files - are stored)

*In many countries, the servers holding the data – the online files with courts’ rulings – are administered by private companies or ministries of justice.*

**VIII. Ethical competences**

*This set of competences covers the code of conduct for judges. The list of ethical competences includes several which might also be covered by the ethical code (competence 53). We have listed the most important of those explicitly in order to obtain information on countries and jurisdictions which do not have ethical codes.*

1. Preparation of the code of conduct

*To what extent do judges, according to the law, take part on the preparation of ethical codes?*

1. Interpretation of the code of conduct (e.g. advising judges on how to behave via "hotlines")

*The existence of such hotlines offering interpretation is typical, for example, for the USA.*

1. Authorization of judges to communicate with media

*Who decides whether and in what capacity judges can communicate with media. This question is included because of a particular interest in those jurisdictions which do not have ethical codes.*

1. Explaining decisions

*Who has the competence to explain decisions (after they have been issued)? This question is included because of a particular interest inf those jurisdictions which do not have ethical codes.*

1. Regulation of off-bench activities – teaching

*Who decides whether judges can teach? This question is included because of a particular interest in those jurisdictions which do not have ethical codes.*

1. Regulation of off-bench activities – party membership

*Who decides whether judges can be members of a political party? This question is included because of a particular interest in those jurisdictions which do not have ethical codes.*

1. Regulation of off-bench activities – political engagement

*Who decides whether judges can have any other political engagement? This question is included because of a particular interest in those jurisdictions which do not have ethical codes.*

1. Regulation of business activities of judges

*Who decides whether judges can engage in business activities? This question is included because of a particular interest in those jurisdictions which do not have ethical codes.*

**References**

- Gutmann, J., Voigt, S. (2018) Judicial Independence in the EU: a puzzle. *European Journal of Law and Economics* 49, 83-100.
- Hanretty, C. (2009) Explaining the De Facto independence of Public Broadcasters*, British Journal of Political Science*, Vol. 40 75–89.
- Hayo, B. and Voigt, S. (2021) Hayo, Bernd and Voigt, Stefan, Judicial Independence: Why Does De Facto Diverge from De Jure? (August 1, 2021). Available at SSRN: https://ssrn.com/abstract=3897343 or <http://dx.doi.org/10.2139/ssrn.3897343>.
- Juwayeyi, M.M. (2017) The Malawi Communications Regulatory Authority: Issues of De Jure and De Facto Independence*. Communication Law and Policy* 22, 213-253.
- Kosař, D. (2018). Beyond Judicial Councils: Forms, Rationales and Impact of Judicial Self Governance in Europe. *German Law Journal* 19, 1567–1612.
- WUTTKE, A. and SCHIMPF (2020), Ch. When the Whole is Greater than the Sum of Its Parts: On the Conceptualization and Measurement of Populist Attitudes and Other Multidimensional Constructs. *American Political Science Review,* Vol. 114, Issue 2, pp. 356-274;

1. In essence, this means that we repeated the very same process twice: first for the internal decision-making processes inside each JSG body, and secondly for the decision-making process for each individual competence. For example, let us presume that in country X judges are selected by a joint decision of the judicial council and the President of the republic. First, the candidate must be approved by the judicial council; then, the President must agree with that selection and appoint the candidate. The judicial council is composed of 10 people, 5 judges and 5 non-judges, and needs a qualified majority to decide. This means that the judicial council is evaluated as 0.5. This judicial council, with 0.5 influence of judges, then adopts a decision together with the minister of justice, which process in fact further dilutes the power directly held by judges. In such a competence the judges’ share of power would equal 0.5 (one cannot take the decision without the other). [↑](#footnote-ref-1)
2. Like our logic implemented in the analysis of the composition of judicial councils, the chosen approach also allows us to forego the agent-principal dilemma, as the inclusion of competences related to the creation of JSG bodies would inevitably lead to the question whether, e.g., court presidents selected and removable by a judge-dominated judicial council behave differently from court presidents selected by ministers of justice. This is an important and relevant question for the interpretation of judicial governance and its relationship to judicial independence in particular. However, it is not a question explored by the JSG Index. [↑](#footnote-ref-2)
